# Four new species of Asian horned toads (Anura, Megophryidae, *Megophrys*) from southern China

**DOI:** 10.3897/zookeys..47983

**Published:** 2020-06-18

**Authors:** Zhi-Tong Lyu, Yuan-Qiu Li, Zhao-Chi Zeng, Jian Zhao, Zu-Yao Liu, Guo-Xin Guo, Ying-Yong Wang

**Affiliations:** 1 State Key Laboratory of Biocontrol/ The Museum of Biology, School of Life Sciences, Sun Yat-sen University, Guangzhou 510275, China; 2 School of Ecology, Sun Yat-sen University, Guangzhou 510006, China; 3 Guangdong Shimentai National Nature Reserve, Qingyuan 513000, China; 4 Shenzhen Shuanghuamu Biological Technology Co., LTD, Shenzhen 51800, China; 5 Institute of Ecology and Evolution, University of Bern, Bern 3012, Switzerland

**Keywords:** cryptic species, diversity, morphology, *
Panophrys
*, taxonomy

## Abstract

Recent phylogenetic analysis encompassing multilocus nuclear-gene and matrilineal mtDNA genealogy has revealed a series of cryptic species of the subgenus Panophrys within genus *Megophrys* from southern and eastern China. This study demonstrates that the *Panophrys* specimens from the hilly areas among Guangdong, Guangxi and Hunan can be morphologically distinguished from all recognized congeners, thereby providing additional supports for the recognitions of four new species of *Panophrys*, namely Megophrys (Panophrys) mirabilis Lyu, Wang & Zhao, **sp. nov.** from northeastern Guangxi, Megophrys (Panophrys) shimentaina Lyu, Liu & Wang, **sp. nov.** from northern Guangdong, and Megophrys (Panophrys) xiangnanensis Lyu, Zeng & Wang, **sp. nov.** and Megophrys (Panophrys) yangmingensis Lyu, Zeng & Wang, **sp. nov.** from southern Hunan. The descriptions of these species take the number of *Megophrys* species to 101, 46 of which belong to the subgenus Panophrys.

## Introduction

The Asian horned toad genus *Megophrys* Kuhl & Van Hasselt, 1822 within the family Megophryidae Bonaparte, 1850, is a typical representative for Oriental fauna, spreading throughout southern China, southern and eastern Himalayas, across Indochina to islands of the Sunda Shelf and the Philippines (Mahony et al. 2017; Liu et al. 2018; Frost 2020). Although morphological identifications on *Megophrys* species are not easy (Li et al. 2014; Liu et al. 2018), with the progress in integrative taxonomy, a large number of new species have been recognized in the last decade, and takes the species number of genus *Megophrys* sensu lato to 97 (Frost 2020).

**Figure 1.**
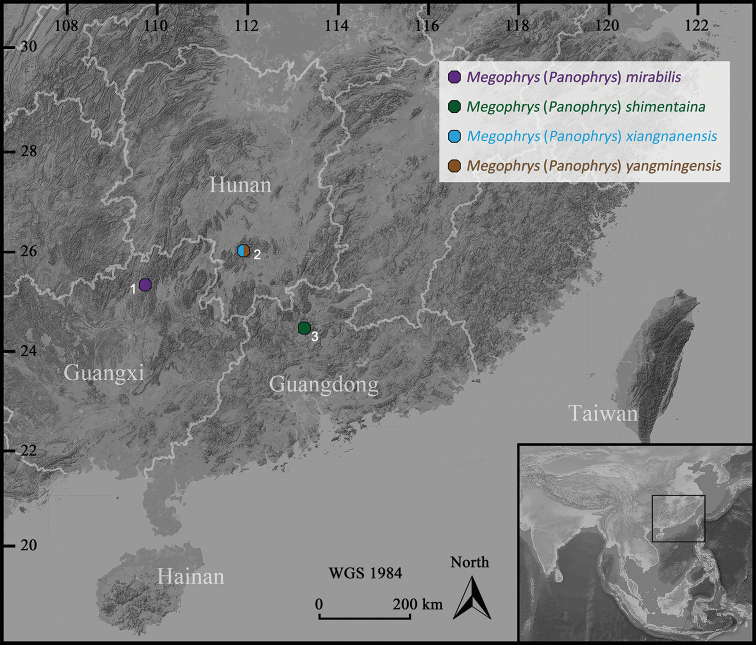
Map showing the collecting location of the new species. **1** Huaping Nature Reserve, Lingui District, Guilin City, Guangxi **2** Mt Yangming, Shuangpai County, Yongzhou City, Hunan **3** Shimentai Nature Reserve, Yingde City, Qingyuan City, Guangdong.

During our herpetological surveys in the hilly areas among Guangdong, Guangxi and Hunan, southern China (Fig. 1), a series of specimens of horned toads were collected. These specimens morphologically belong to genus *Megophrys* but could not be assigned to any recognized species by the combinations of characteristics. Furthermore, the phylogenetic analysis encompassing multilocus nuclear-gene and matrilineal mtDNA genealogy conducted by Liu et al. (2018) has indicated that these specimens should be regarded as four cryptic species of the subgenus Panophrys, i.e., *M.* sp29 from northern Guangdong, *M.* sp25 from northeastern Guangxi, and *M.* sp2 and *M.* sp28 from southwestern Hunan. In this study, as a follow-up work on this series of specimens, we provide the additional morphological comparisons and descriptions to substantiate the recognition of these four cryptic species of *Panophrys* from southern China.

## Materials and methods

**Taxonomic system.** The higher systematics of Asian horned toads has been in intensive debates for decades (Delorme et al. 2006; Frost et al. 2006; Li and Wang 2008; Chen et al. 2017; Deuti et al. 2017; Mahony et al. 2017; Liu et al. 2018; Li et al. 2020). In this study, not involving in the controversy of generic relationship in subfamily Megophryinae, we followed the most recent taxonomic arrangement (Mahony et al. 2017; Liu et al. 2018; Frost 2020), in which the genus *Megophrys* is considered to include seven subgenera: *Atympanophrys* Tian & Hu, 1983, *Brachytarsophrys* Tian & Hu, 1983, *Megophrys*, *Ophryophryne* Boulenger, 1903, *Pelobatrachus* Beddard, 1908, *Panophrys* Rao & Yang, 1997, and Xenophrys Günther, 1864. Since the subgenus Panophrys has been unanimously considered as a monophyletic group that is significantly divergent from other subgenera, we perform the analyses and comparisons on the undescribed specimens with *Panophrys* congeners in this study.

**Phylogeny.** Two mitochondrial genes, namely partial 16S ribosomal RNA gene (16S) and partial cytochrome C oxidase 1 gene (CO1), were used for phylogenetic analysis. All sequences were attained from GenBank, encompassing 17 samples of the unnamed species (originally submitted by Liu et al. 2018) and 40 samples from 40 recognized Panophrys congeners. Besides, two samples of subgenus Xenophrys were incorporated into our dataset as out-groups. Detailed information of these materials is given in Table 1.

**Table 1.** Localities, voucher information, and GenBank numbers for all samples used in this study.

ID	Species	Localities	Voucher number	16s	co1
**Megophrys (Panophrys)**					
**1**	M. (Pa.) mirabilis sp. nov.	China: Guangxi: Huaping Nature Reserve	SYS a002192	MH406669	MH406109
**2**	M. (Pa.) mirabilis sp. nov.	China: Guangxi: Huaping Nature Reserve	SYS a002193	MH406670	MH406110
**3**	M. (Pa.) mirabilis sp. nov.	China: Guangxi: Huaping Nature Reserve	SYS a002289	MH406681	MH406127
**4**	M. (Pa.) mirabilis sp. nov.	China: Guangxi: Huaping Nature Reserve	SYS a002917	MH406724	MH406176
**5**	M. (Pa.) shimentaina sp. nov.	China: Guangdong: Shimentai Nature Reserve	SYS a002077	MH406655	MH406092
**6**	M. (Pa.) shimentaina sp. nov.	China: Guangdong: Shimentai Nature Reserve	SYS a002081	MH406656	MH406093
**7**	M. (Pa.) shimentaina sp. nov.	China: Guangdong: Shimentai Nature Reserve	SYS a004172	MH406787	MH406249
**8**	M. (Pa.) shimentaina sp. nov.	China: Guangdong: Shimentai Nature Reserve	SYS a004173	MH406788	MH406250
**9**	M. (Pa.) xiangnanensis sp. nov.	China: Hunan: Mt Yangming	SYS a002874	MH406713	MH406165
**10**	M. (Pa.) xiangnanensis sp. nov.	China: Hunan: Mt Yangming	SYS a002875	MH406714	MH406166
**11**	M. (Pa.) xiangnanensis sp. nov.	China: Hunan: Mt Yangming	SYS a002876	MH406715	MH406167
**12**	M. (Pa.) xiangnanensis sp. nov.	China: Hunan: Mt Yangming	SYS a002878	MH406717	MH406169
**13**	M. (Pa.) xiangnanensis sp. nov.	China: Hunan: Mt Yangming	SYS a002879	MH406718	MH406170
**14**	M. (Pa.) yangmingensis sp. nov.	China: Hunan: Mt Yangming	SYS a002877	MH406716	MH406168
**15**	M. (Pa.) yangmingensis sp. nov.	China: Hunan: Mt Yangming	SYS a002888	MH406719	MH406171
**16**	M. (Pa.) yangmingensis sp. nov.	China: Hunan: Mt Yangming	SYS a002889	MH406720	MH406172
**17**	M. (Pa.) yangmingensis sp. nov.	China: Hunan: Mt Yangming	SYS a002890	MH406721	MH406173
**18**	M. (Pa.) acuta	China: Guangdong: Heishiding Nature Reserve	SYS a002266	KJ579119	MH406122
**19**	M. (Pa.) baolongensis	China: Chongqing: Baolong Town	KIZ 019216	KX811813	KX812093
**20**	M. (Pa.) binchuanensis	China: Yunnan: Mt. Jizu	KIZ 019441	KX811849	KX812112
**21**	M. (Pa.) binlingensis	China: Sichuan: Mt. Wawu	SYS a005313	MH406892	MH406354
**22**	M. (Pa.) boettgeri	China: Fujian: Mt. Wuyi	SYS a004149	MF667878	MH406247
**23**	M. (Pa.) brachykolos	China: Hong Kong	SYS a002258	KJ560403	MH406120
**24**	M. (Pa.) caudoprocta	China: Hunan: Badagongshan Nature Reserve	SYS a004281	MH406795	MH406257
**25**	M. (Pa.) cheni	China: Hunan: Taoyuandong Nature Reserve	SYS a002142	KJ560398	MH406098
**26**	M. (Pa.) daweimontis	China: Yunnan: Mt. Dawei	KIZ 048997	KX811867	KX812125
**27**	M. (Pa.) dongguanensis	China: Guangdong: Mt. Yinping	SYS a001973	MH406647	MH406083
**28**	M. (Pa.) fansipanensis	Vietnam: Lao Cai: Sa Pa	VNMN 2018.01	MH514886	/
**29**	M. (Pa.) hoanglienensis	Vietnam: Lao Cai: Sa Pa	VNMN 07034	MH514890	/
**30**	M. (Pa.) huangshanensis	China: Anhui: Mt. Huangshan	SYS a002703	MF667883	MH406161
**31**	M. (Pa.) insularis	China: Guangdong: Nan’ao Island	SYS a002169	MF667887	MF667924
**32**	M. (Pa.) jiangi	China: Guizhou: Kuankuoshui Nature Reserve	CIB KKS20180722006	MN107743	MN107748
**33**	M. (Pa.) jingdongensis	China: Yunnan: Mt. Wuliang	SYS a003928	MH406773	MH406232
**34**	M. (Pa.) jinggangensis	China: Jiangxi: Mt. Jinggang	SYS a004028	MH406780	MH406239
**35**	M. (Pa.) jiulianensis	China: Jiangxi: Mt. Jiulian	SYS a004219	MH406791	MH406253
**36**	M. (Pa.) kuatunensis	China: Fujian: Mt. Wuyi	SYS a003449	MF667881	MH406206
**37**	M. (Pa.) leishanensis	China: Guizhou: Mt. Leigong	SYSa002213	MH406673	MH406113
**38**	M. (Pa.) liboensis	China: Guizhou: Libo Country	20150813001	MF285253	/
**39**	M. (Pa.) lini	China: Hunan: Taoyuandong Nature Reserve	SYS a002381	MF667874	MH406135
**40**	M. (Pa.) lishuiensis	China: Zhejiang: Lishui City	WYF00169	KY021418	/
**41**	M. (Pa.) minor	China: Sichuan: Mt. Qingcheng	SYS a003209	MF667862	MH406194
**42**	M. (Pa.) mufumontana	China: Hunan: Mt. Mufu	SYS a006390	MK524104	MK524135
**43**	M. (Pa.) nankunensis	China: Guangdong: Mt. Nankun	SYS a004501	MH406822	MH406284
**44**	M. (Pa.) nanlingensis	China: Guangdong: Nanling Nature Reserve	SYS a001964	MH406646	MH406082
**45**	M. (Pa.) obesa	China: Guangdong: Heishiding Nature Reserve	SYS a002271	KJ579121	MH406123
**46**	M. (Pa.) ombrophila	China: Fujian: Mt. Wuyi	WUYI2015101	KX856397	/
**47**	M. (Pa.) omeimontis	China: Sichuan: Mt. Emei	SYS a005301	MH406887	MH406349
**48**	M. (Pa.) palpebralespinosa	Vietnam: Thanh Hoa: Pu Hu Nature Reserve	KIZ 011650	KX811889	KX812138
**49**	M. (Pa.) rubrimera	Vietnam: Lao Cai: Sa Pa	VNMN 2017.002	MF536420	/
**50**	M. (Pa.) sangzhiensis	China: Hunan: Badagongshan Nature Reserve	SYS a004306	MH406797	MH406259
**51**	M. (Pa.) shunhuangensis	China: Hunan: Nanshan Forest Park	HNNU 18NS01	MK836023	MK977594
**52**	M. (Pa.) spinata	China: Guizhou: Mt. Leigong	SYS a002226	MH406675	MH406115
**53**	M. (Pa.) tuberogranulatus	China: Hunan: Badagongshan Nature Reserve	SYS a004310	MH406801	MH406263
**54**	M. (Pa.) wugongensis	China: Jiangxi: Mt. Wugong	SYS a004800	MH406853	MH406315
**55**	M. (Pa.) wuliangshanensis	China: Yunnan: Mt. Wuliang	SYS a003924	MH406771	MH406230
**56**	M. (Pa.) wushanensis	China: Hubei: Shennongjia Nature Reserve	SYS a003008	MH406732	MH406184
**57**	M. (Pa.) xianjuensis	China: Zhejiang: Xianju County	CIB XJ190505	MN563753	MN563769
**Megophrys (Xenophrys)**					
**58**	M. (X.) glandulosa	China: Yunnan: Mt. Gaoligong	SYS a003758	MH406755	MH406214
**59**	M. (X.) mangshanensis	China: Guangdong: Mt. Sanyue	SYS a002177	MH406666	MH406106

DNA sequences were aligned by the Clustal W algorithm with default parameters (Thompson et al. 1997) and trimmed with the gaps partially deleted in MEGA 6 (Tamura et al. 2013). Two gene segments, 632 base pairs (bp) of CO1 and 541 bp of16S, were concatenated seriatim into a 1173-bp sequence, and were further tested in jmodeltest v2.1.2 with Akaike and Bayesian information criteria, all resulting the best-fitting nucleotide substitution models of GTR+I+G. Sequenced data was analyzed using Bayesian inference (BI) in MrBayes 3.2.4 (Ronquist et al. 2012). Two independent runs were conducted in a BI analysis, each of which was performed for 10,000,000 generations and sampled every 1000 generations with the first 25% samples were discarded as burn-in, resulting a potential scale reduction factor (PSRF) of < 0.005. Mean genetic distances of 16S gene between and within species were calculated in MEGA 6 using the uncorrected *p*-distance model.

**Bioacoustics.** Advertisement calls of the unnamed species were recorded in the field by a SONY PCM-D50 digital sound recorder. The sound files in wave format were sampled at 48 kHz with 24 bits in depth. Raven pro 1.5 (Cornell Lab of Ornithology, 2003–2014) was used to output the spectrograms and to measure interrelated parameters with Fast Fourier transform of 256 points and a 50% overlap. The following measurements were performed: call/note duration (the difference between begin time and end time for a selected call/note), notes per call, inter-note intervals (the difference between end time for a selected note and begin time for the next selected note), peak frequency (the frequency at which peak power occurs within the selected call), high frequency (the highest frequency of the selected call), low frequency (the lowest frequency of the selected call), bandwidth 90% (the difference between the 5% and 95% frequencies of a selected call).

**Morphology.** Thirty-six unnamed specimens from the hilly areas among Guangdong, Guangxi and Hunan, southern China were examined, 17 of which have been used in the phylogenetic analysis. All examined specimens were fixed in 10% buffered formalin and later transferred to 70% ethanol. All studied specimens are deposited in The Museum of Biology, Sun Yat-sen University (**SYS**), and Chengdu Institute of Biology, Chinese Academy of Sciences (**CIB**), China.

External measurements were made for the unnamed specimens with digital calipers (Neiko 01407A Stainless Steel 6-Inch Digital Caliper, USA) to the nearest 0.1 mm. Mean and standard deviation (SD) were calculated in R 3.3.2 (R Core Team 2016). These measurements were as follows:

**ED** eye diameter (from the anterior corner of the eye to posterior corner of the eye);

**FTL** foot length (from distal end of shank to the tip of digit IV);

**HDL** head length (from tip of snout to the articulation of the jaw);

**HDW** head width (head width at the commissure of the jaws);

**HND** hand length (from the proximal border of the outer palmar tubercle to the tip of digit III);

**IND** internasal distance (distance between nares);

**IOD** interorbital distance (minimum distance between upper eyelids);

**RAD** radio-ulna length (from the flexed elbow to the proximal border of the outer palmar tubercle);

**SNT** snout length (from tip of snout to the anterior corner of the eye);

**SVL** snout-vent length (from tip of snout to posterior margin of vent);

**TD** tympanum diameter (horizontal diameter of tympanum);

**TED** tympanum-eye distance (from anterior edge of tympanum to posterior corner of the eye);

**TIB** tibial length (from the outer surface of the flexed knee to the heel).

Sex was determined by secondary sexual characters, i.e., the presence of vocal sac, nuptial pads/spines in males (Fei et al. 2016).

Morphological characters of all 42 recognized congeners of subgenus Panophrys for comparisons were based on the examination of museum specimens listed in Appendix I and on information available in the literature (Table 2).

**Table 2.** Literature for morphological characters of 42 recognized species of Megophrys (Panophrys).

ID	Species	References
**1**	M. (Pa.) acuta Wang, Li & Jin, 2014	Li et al. 2014
**2**	M. (Pa.) baolongensis Ye, Fei & Xie, 2007	Ye et al. 2007; Fei and Ye 2016
**3**	M. (Pa.) binchuanensis Ye & Fei, 1995	Fei and Ye 2016
**4**	M. (Pa.) binlingensis Jiang, Fei & Ye, 2009	Fei and Ye 2016
**5**	M. (Pa.) boettgeri (Boulenger, 1899)	Fei and Ye 2016
**6**	M. (Pa.) brachykolos Inger & Romer, 1961	Fei and Ye 2016
**7**	M. (Pa.) caudoprocta Shen, 1994	Fei and Ye 2016
**8**	M. (Pa.) cheni (Wang & Liu, 2014)	Wang et al. 2014
**9**	M. (Pa.) daweimontis Rao & Yang, 1997	Fei and Ye 2016
**10**	M. (Pa.) dongguanensis Wang & Wang	Wang et al. 2019a
**11**	M. (Pa.) fansipanensis Tapley, Cutajar, Mahony, Nguyen, Dau, Luong, Le, Nguyen, Nguyen, Portway, Luong & Rowley, 2018	Tapley et al. 2018
**12**	M. (Pa.) hoanglienensis Tapley, Cutajar, Mahony, Nguyen, Dau, Luong, Le, Nguyen, Nguyen, Portway, Luong & Rowley, 2018	Tapley et al. 2018
**13**	M. (Pa.) huangshanensis Fei & Ye, 2005	Fei and Ye 2016
**14**	M. (Pa.) insularis (Wang, Liu, Lyu, Zeng & Wang, 2017)	Wang et al. 2017a
**15**	M. (Pa.) jiangi Liu, Li, Wei, Xu, Cheng, Wang & Wu, 2020	Liu et al. 2020
**16**	M. (Pa.) jingdongensis Fei & Ye, 1983	Fei and Ye 2016
**17**	M. (Pa.) jinggangensis (Wang, 2012)	Wang et al. 2012
**18**	M. (Pa.) jiulianensis Wang, Zeng, Lyu & Wang	Wang et al. 2019a
**19**	M. (Pa.) kuatunensis Pope, 1929	Fei and Ye 2016
**20**	M. (Pa.) latidactyla Orlov, Poyarkov & Nguyen, 2015	Orlov et al. 2015
**21**	M. (Pa.) leishanensis Li, Xu, Liu, Jiang, Wei & Wang, 2018	Li et al. 2018
**22**	M. (Pa.) liboensis (Zhang, Li, Xiao, Li, Pan, Wang, Zhang & Zhou, 2017)	Zhang et al. 2017
**23**	M. (Pa.) lini (Wang & Yang, 2014)	Wang et al. 2014
**24**	M. (Pa.) lishuiensis (Wang, Liu & Jiang, 2017)	Wang et al. 2017b
**25**	M. (Pa.) minor Stejneger, 1926	Fei and Ye 2016
**26**	M. (Pa.) mufumontana Wang, Lyu & Wang	Wang et al. 2019a
**27**	M. (Pa.) nankunensis Wang, Zeng & Wang	Wang et al. 2019a
**28**	M. (Pa.) nanlingensis Lyu, Wang, Liu & Wang	Wang et al. 2019a
**29**	M. (Pa.) obesa Wang, Li & Zhao, 2014	Li et al. 2014
**30**	M. (Pa.) ombrophila Messenger & Dahn, 2019	Messenger et al. 2019
**31**	M. (Pa.) omeimontis Liu, 1950	Fei and Ye 2016
**32**	M. (Pa.) palpebralespinosa Bourret, 1937	Fei and Ye 2016
**33**	M. (Pa.) robrimera Tapley, Cutajar, Mahony, Chung, Dau, Nguyen, Luong & Rowley, 2017	Tapley et al. 2017
**34**	M. (Pa.) sangzhiensis Jiang, Ye & Fei, 2008	Jiang et al. 2008; Fei and Ye 2016
**35**	M. (Pa.) shuichengensis Tian & Sun, 1995	Tian et al. 2000; Fei and Ye 2016
**36**	M. (Pa.) shunhuangensis Wang, Deng, Liu, Wu & Liu, 2019	Wang et al. 2019b
**37**	M. (Pa.) spinata Liu & Hu, 1973	Fei and Ye 2016
**38**	M. (Pa.) tuberogranulatus Shen, Mo & Li, 2010	Mo et al. 2010; Fei and Ye 2016
**39**	M. (Pa.) wugongensis Wang, Lyu & Wang	Wang et al. 2019a
**40**	M. (Pa.) wuliangshanensis Ye & Fei, 1995	Fei and Ye 2016
**41**	M. (Pa.) wushanensis Ye & Fei, 1995	Fei and Ye 2016
**42**	M. (Pa.) xianjuensis Wang, Wu, Peng, Shi, Lu & Wu, 2020	Wang et al. 2020

## Results

**Figure 2.**
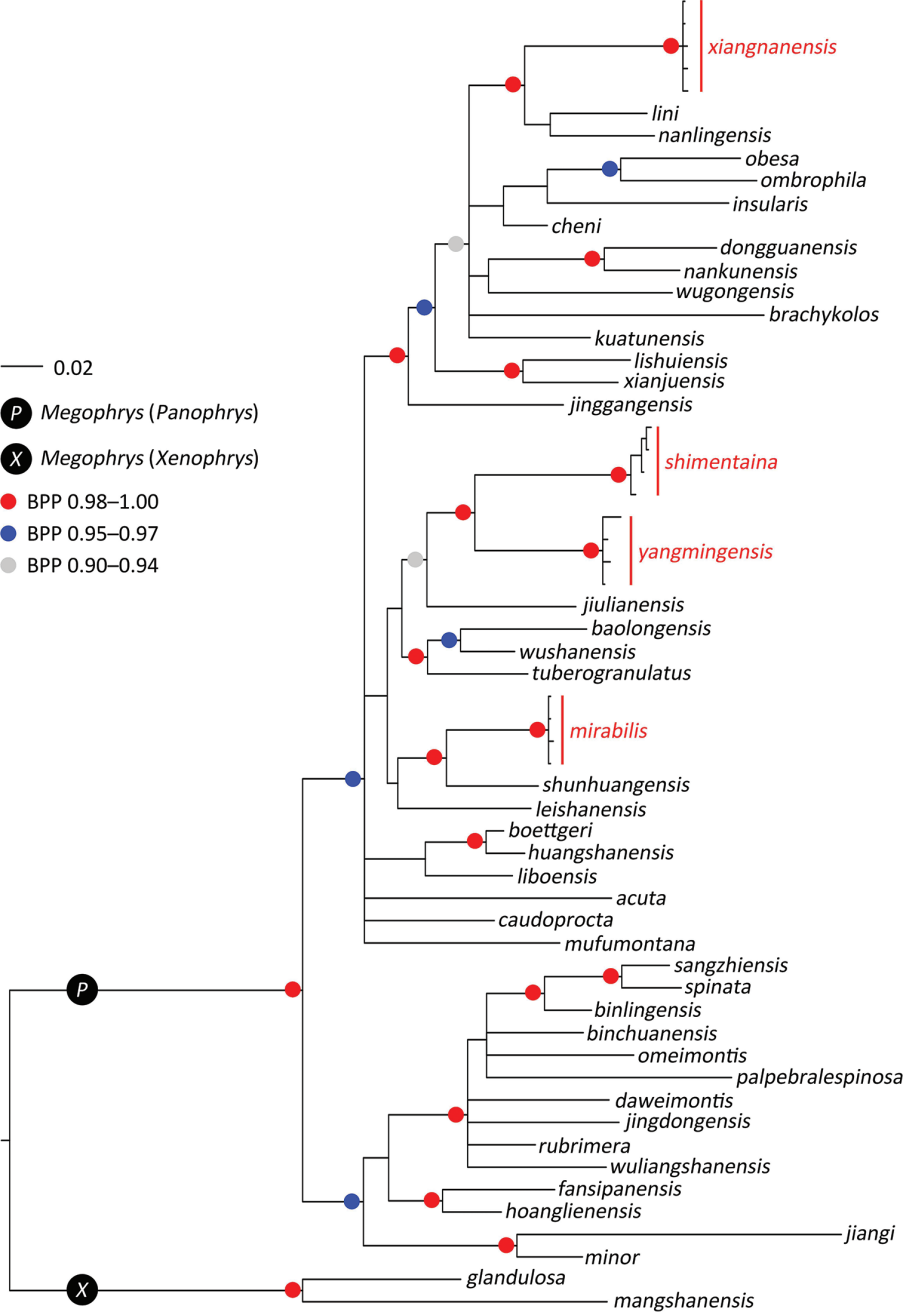
Phylogenetic tree of Megophrys (Panophrys) inferred from mitochondrial genes by Bayesian inference.

The BI phylogenetic result is shown in Fig. 2 with Bayesian posterior probabilities (BPP) for major nodes > 0.90. The mean *p*-distances of 16S gene among all in-group and out-group species used in this study are given in Table 3. The diagnostic characters separating all 42 recognized species of the subgenus Panophrys are given in Table 4.

The unnamed samples from Huaping Nature Reserve, Guangxi (samples ID 1–4 in Table 1), are clustered into a monophyletic clade with strong node supports (BPP 1.00) and almost have no molecular divergences (*p*-distances 0.0), which was defined as a cryptic species *Megophrys* sp25 in Liu et al. (2018); this population can be further distinguished from all recognized and undescribed species by a combination of distinctive morphological characters (see Taxonomic accounts below). Therefore, the population from Huaping Nature Reserve represents a separately evolving lineage, and is described as a new species, Megophrys (Panophrys) mirabilis sp. nov.

The samples from Shimentai Nature Reserve, Guangxi (samples ID 5–8 in Table 1), are grouped into a monophyletic clade with strong node supports (BPP 1.00) and almost have no molecular divergences (*p*-distances 0.0), which was defined as a cryptic species *Megophrys* sp29 in Liu et al. (2018); samples (ID 14–17 in Table 1) from Mt Yangming, Hunan, are clustered into a monophyletic clade with strong node supports (BPP 1.00) and have small molecular divergences (*p*-distances 0.3), which was defined as a cryptic species *M.* sp28 in Liu et al. (2018). These two populations are sister taxa to each other with significant genetic divergences (*p*-distances 4.1), and can be distinguished from all congeners by a combination of distinctive morphological characters (see Taxonomic accounts below). Therefore, the populations from Shimentai Nature Reserve and Mt Yangming represent two separately evolving lineage, and are described as new species, Megophrys (Panophrys) shimentaina sp. nov. and Megophrys (Panophrys) yangmingensis sp. nov., respectively.

The other samples from Mt Yangming, Hunan (samples ID 9–13 in Table 1), cluster into a monophyletic clade with strong node supports (BPP 1.00) and almost have no molecular divergences (*p*-distances 0.0), which was defined as a cryptic species *Megophrys* sp2 in Liu et al. (2018). This clade is conspicuously distant from the sympatric species Megophrys (Panophrys) yangmingensis sp. nov. in phylogeny. Furthermore, this population can be distinguished from all congener species by a combination of distinctive morphological characters (see Taxonomic accounts below). Therefore, this population from Mt Yangming represents a separately evolving lineage, and is described as a new species, Megophrys (Panophrys) xiangnanensis sp. nov.

**Table 3.** Mean *p*-distance of 16S gene among the Megophrys (Panophrys) species used in this study.

ID	Species	1– 4	5– 8	9– 13	14–17	18	19	20	21	22	23	24	25	26	27	28	29	30	31	32	33	34	35	36	37	38	39	40	41	42	43	44	45	46	47	48	49	50	51	52	53	54	55	56	57
**1– 4**	M. (Pa.) mirabilis sp. nov.	0.0																																											
**5– 8**	M. (Pa.) shimentaina sp. nov.	3.8	0.0																																										
**9– 13**	M. (Pa.) xiangnanensis sp. nov.	3.9	4.1	0.0																																									
**14–17**	M. (Pa.) yangmingensis sp. nov.	3.4	3.4	4.3	0.3																																								
**18**	M. (Pa.) acuta	7.3	8.8	7.3	7.3	/																																							
**19**	M. (Pa.) baolongensis	3.6	4.0	4.4	2.9	7.3	/																																						
**20**	M. (Pa.) binchuanensis	4.8	5.4	4.8	4.9	8.1	4.4	/																																					
**21**	M. (Pa.) binlingensis	4.9	5.1	5.0	4.3	8.9	3.8	2.5	/																																				
**22**	M. (Pa.) boettgeri	2.4	3.4	3.9	2.1	7.0	2.7	4.0	3.7	/																																			
**23**	M. (Pa.) brachykolos	5.4	5.5	3.9	5.2	8.1	4.6	5.3	6.5	4.7	/																																		
**24**	M. (Pa.) caudoprocta	3.5	4.3	5.0	3.2	7.3	2.5	4.4	3.7	2.4	5.8	/																																	
**25**	M. (Pa.) cheni	3.5	4.0	3.0	3.5	7.8	2.7	3.8	4.3	2.6	4.3	3.9	/																																
**26**	M. (Pa.) daweimontis	4.4	5.4	4.2	4.4	7.0	4.0	2.8	2.9	3.4	6.3	4.0	3.6	/																															
**27**	M. (Pa.) dongguanensis	5.2	4.7	4.3	4.9	8.1	4.0	5.3	5.2	4.1	5.8	5.0	2.8	4.6	/																														
**28**	M. (Pa.) fansipanensis	5.1	5.0	4.7	4.6	10.5	4.0	4.6	4.2	4.2	5.5	4.6	4.4	4.6	5.7	/																													
**29**	M. (Pa.) hoanglienensis	4.6	5.0	4.7	4.0	8.4	3.8	3.2	2.9	3.6	5.7	4.0	4.0	3.6	4.9	2.5	/																												
**30**	M. (Pa.) huangshanensis	2.8	3.8	4.3	2.6	7.0	3.0	4.4	4.1	0.7	5.0	2.8	3.0	3.6	4.5	4.6	4.0	/																											
**31**	M. (Pa.) insularis	5.2	4.5	4.1	4.5	9.2	4.2	5.7	5.2	4.5	5.6	5.2	2.8	5.5	4.3	4.5	4.7	4.9	/																										
**32**	M. (Pa.) jiangi	7.3	8.3	7.9	7.5	12.0	7.6	8.4	8.7	7.1	8.6	8.1	7.7	8.2	8.8	8.0	7.8	7.9	8.6	/																									
**33**	M. (Pa.) jingdongensis	4.5	5.1	4.9	4.7	8.6	4.2	2.9	2.6	4.1	6.7	3.7	4.1	2.5	6.0	4.0	4.0	4.5	5.8	8.6	/																								
**34**	M. (Pa.) jinggangensis	3.5	4.9	3.0	3.7	7.3	3.6	4.2	4.1	3.2	5.0	4.3	3.0	4.0	5.0	4.6	4.4	3.6	4.1	7.0	4.3	/																							
**35**	M. (Pa.) jiulianensis	2.8	3.8	4.3	2.8	8.1	2.9	4.9	4.3	2.2	5.2	3.0	3.5	4.8	4.5	3.8	3.2	2.6	4.5	8.1	5.0	3.9	/																						
**36**	M. (Pa.) kuatunensis	3.7	3.8	3.4	3.6	8.1	3.0	4.6	4.7	2.8	4.1	3.7	2.4	4.0	2.8	4.4	4.2	3.2	3.4	8.1	4.9	3.7	3.6	/																					
**37**	M. (Pa.) leishanensis	2.4	3.6	4.1	2.6	7.3	2.5	3.4	3.4	1.7	4.9	2.8	3.0	3.4	4.7	3.6	3.4	2.1	4.7	6.6	3.5	3.4	2.4	3.4	/																				
**38**	M. (Pa.) liboensis	3.4	3.8	4.7	3.2	7.8	3.2	4.9	4.9	1.7	4.6	3.2	3.5	4.4	4.7	4.7	4.2	2.1	5.0	7.7	5.2	4.3	3.0	3.0	2.8	/																			
**39**	M. (Pa.) lini	4.1	4.7	2.2	4.5	7.0	4.7	4.9	5.2	3.4	3.9	4.7	3.0	4.4	4.1	5.5	5.3	3.7	4.3	8.1	5.0	3.6	4.5	3.2	3.9	4.3	/																		
**40**	M. (Pa.) lishuiensis	5.0	6.9	5.8	5.0	8.4	4.5	6.3	6.3	3.8	6.8	5.3	4.8	6.0	5.8	6.5	5.3	4.8	5.3	10.6	7.8	6.0	4.8	4.5	4.5	4.5	6.0	/																	
**41**	M. (Pa.) minor	5.6	6.2	6.2	5.1	9.5	6.1	5.5	5.6	4.5	6.4	5.6	5.4	5.1	6.4	5.5	5.1	5.1	7.1	7.0	5.8	5.4	6.0	5.4	5.1	5.4	6.2	7.6	/																
**42**	M. (Pa.) mufumontana	3.8	4.2	4.7	2.6	8.6	3.2	4.6	4.3	2.4	5.4	3.2	3.6	4.4	5.1	4.5	4.0	3.0	4.5	7.2	4.7	3.4	3.2	3.4	2.6	3.4	4.9	5.0	5.5	/															
**43**	M. (Pa.) nankunensis	4.5	4.3	4.1	4.3	8.9	3.2	5.5	5.0	3.9	5.4	4.3	2.2	4.6	2.6	5.3	5.1	4.3	4.1	8.6	5.2	4.5	4.3	3.0	4.1	4.7	4.3	5.8	6.7	4.5	/														
**44**	M. (Pa.) nanlingensis	4.9	5.8	3.4	4.9	7.8	5.3	5.7	5.2	4.3	5.0	6.0	3.9	5.3	4.9	5.5	5.3	4.7	4.5	8.5	6.0	4.7	5.0	4.5	4.5	5.8	3.6	6.3	6.0	5.3	5.0	/													
**45**	M. (Pa.) obesa	6.2	7.1	5.4	6.8	8.4	6.8	7.8	8.1	5.1	7.8	7.6	3.2	5.9	5.1	7.6	7.6	5.7	5.4	11.7	7.0	6.5	6.8	4.6	5.9	6.5	5.1	5.7	9.2	7.6	4.9	6.5	/												
**46**	M. (Pa.) ombrophila	5.2	5.8	5.0	5.2	9.7	4.9	5.9	6.2	4.5	6.0	6.0	3.2	5.3	4.7	5.5	5.5	4.9	4.5	8.4	6.0	5.2	5.2	4.7	4.3	5.6	5.2	4.8	7.1	5.4	4.7	6.0	4.1	/											
**47**	M. (Pa.) omeimontis	4.7	5.1	3.9	4.5	9.2	4.6	2.7	2.4	3.9	5.8	4.3	3.9	2.7	5.0	3.6	3.0	4.3	5.2	9.0	2.4	4.5	4.5	4.3	3.6	4.7	4.5	6.1	5.6	4.5	4.7	5.2	6.8	5.4	/										
**48**	M. (Pa.) palpebralespinosa	5.7	6.7	5.5	6.1	8.4	4.9	3.6	4.0	4.9	6.6	4.8	5.1	3.6	6.3	6.1	5.1	4.9	7.0	9.5	4.4	5.7	6.3	5.1	4.4	5.9	4.7	7.3	5.7	5.9	6.1	6.3	9.2	6.8	4.0	/									
**49**	M. (Pa.) rubrimera	4.7	5.4	5.1	4.2	9.7	4.2	2.5	2.7	2.8	5.9	4.2	3.8	2.3	5.5	4.2	3.6	3.6	5.7	8.2	2.3	4.2	4.2	4.2	3.2	4.2	5.3	6.5	5.1	3.8	4.9	5.7	7.3	5.5	2.5	4.4	/								
**50**	M. (Pa.) sangzhiensis	5.2	5.6	5.2	4.9	9.2	4.4	3.4	1.7	4.3	7.1	4.3	4.5	3.0	5.4	4.2	3.6	4.7	5.2	9.4	3.5	4.9	4.9	5.2	3.9	5.4	5.4	6.5	6.2	5.1	5.0	5.4	7.8	6.2	3.7	5.3	3.6	/							
**51**	M. (Pa.) shunhuangensis	1.5	3.4	3.4	2.4	5.9	2.5	4.0	4.3	1.7	4.1	3.0	2.6	4.0	4.3	4.0	3.4	2.1	4.1	6.8	4.5	3.0	2.2	2.6	1.7	2.0	3.4	3.8	4.9	2.6	3.5	4.1	5.7	4.5	4.1	5.3	4.0	4.7	/						
**52**	M. (Pa.) spinata	4.9	5.1	4.7	4.5	9.2	4.4	3.2	1.1	3.9	6.7	4.3	4.5	3.2	5.4	4.2	3.2	3.9	5.6	9.4	3.4	4.9	4.5	5.2	3.5	5.0	4.7	6.5	5.8	4.9	5.4	5.0	7.8	6.2	2.8	4.6	3.4	1.7	4.3	/					
**53**	M. (Pa.) tuberogranulatus	2.6	3.4	3.7	2.2	7.0	1.7	3.6	3.0	1.7	4.7	2.4	2.2	3.4	3.9	4.0	3.4	2.1	3.9	7.7	3.4	2.6	2.4	2.6	1.9	2.8	3.9	4.3	5.1	2.6	3.0	4.5	5.1	4.3	3.9	4.8	3.2	3.2	2.1	3.5	/				
**54**	M. (Pa.) wugongensis	3.9	4.2	3.9	3.9	8.9	4.2	4.6	4.5	3.6	6.0	4.7	2.8	4.0	4.3	4.2	4.0	3.7	4.5	8.1	4.3	3.6	3.4	4.3	3.7	4.7	4.5	6.0	5.3	4.9	4.3	4.5	5.7	4.9	4.5	6.5	4.2	4.5	3.7	4.1	3.4	/			
**55**	M. (Pa.) wuliangshanensis	3.9	5.5	4.7	4.9	9.2	4.6	3.2	3.4	4.1	6.3	4.5	4.5	2.9	5.8	4.7	4.6	4.5	5.8	8.3	3.0	4.7	4.7	4.3	3.7	4.9	5.4	6.5	5.4	4.7	5.2	6.2	7.0	5.8	3.0	4.6	2.8	4.3	3.7	3.7	3.7	4.9	/		
**56**	M. (Pa.) wushanensis	3.6	3.6	3.6	2.8	8.1	2.1	4.0	3.9	2.6	4.9	3.4	3.2	3.6	3.9	3.8	3.6	3.0	3.6	7.7	4.3	3.2	3.0	3.2	2.4	3.4	4.5	4.0	6.0	3.2	3.7	5.0	6.0	4.3	3.7	5.0	3.8	4.5	2.6	4.5	1.7	3.9	3.9	/	
**57**	M. (Pa.) xianjuensis	3.6	4.4	4.0	3.2	7.3	3.2	4.0	4.4	2.3	4.9	3.6	3.0	3.6	4.2	4.8	3.8	3.0	4.2	7.6	4.4	4.0	3.4	3.0	2.7	3.2	4.6	2.8	5.5	3.4	4.0	5.1	5.1	4.6	3.8	5.1	3.8	5.3	2.9	5.0	2.9	4.8	4.4	3.2	/

**Table 4.** Diagnostic characters separating all 46 species of the Megophrys (Panophrys).

ID	Species	SVL in males (in mm)	SVL in females (in mm)	Horn-like tubercle at upper eyelid: slightly large (2), small (1)	Vomerine teeth: present (1), or absent (0)	Tongue: notched (1), or not notched (0)	Lateral fringes on toes: wide (2), narrow (1), or lacking (0)	Webs on toes: more than one-fourth (2), rudimentary (1), or lacking (0)	TD/ED	TIB/SVL
**1**	M. (Pa.) mirabilis sp. nov.	55.8–61.4	68.5–74.8	2	0	0	1	1	0.49–0.63	0.45–0.47
**2**	M. (Pa.) shimentaina sp. nov.	28.0–30.6	/	1	1	0	1	1	0.57–0.66	0.44–0.53
**3**	M. (Pa.) xiangnanensis sp. nov.	38.6–42.0	44.4	1	0	0	2	1	0.38–0.49	0.41–0.46
**4**	M. (Pa.) yangmingensis sp. nov.	33.2–37.1	45.2	1	0	0	1	1	0.42–0.50	0.44–0.51
**5**	M. (Pa.) acuta	27.1–33.0	28.1–33.6	2	0	0	1	1	0.57–0.71	0.38–0.45
**6**	M. (Pa.) baolongensis	42.0–45.0	/	1	0	1	0	0	0.41	0.46
**7**	M. (Pa.) binchuanensis	32.0–36.0	40.2–42.5	1	0	1 or 0	2	1	0.33–0.50	0.46–0.48
**8**	M. (Pa.) binlingensis	45.1–51.0	/	1	0	1	/	1	0.47–0.52	0.52–0.53
**9**	M. (Pa.) boettgeri	34.5–37.8	39.7–46.8	1	0	1	2	1	0.40–0.67	0.45–0.49
**10**	M. (Pa.) brachykolos	33.7–39.3	33.9–45.9	1	0	0	0	1	> 0.50	0.37–0.42
**11**	M. (Pa.) caudoprocta	81.3	/	2	1	0	/	1	0.5	0.51
**12**	M. (Pa.) cheni	26.2–29.5	31.8–34.1	1	0	1	2	1	0.41–0.54	0.50–0.54
**13**	M. (Pa.) daweimontis	34.0–37.0	40.0–46.0	1	1	/	0	0	/	0.54
**14**	M. (Pa.) dongguanensis	30.2–39.3	/	1	1	0	0	1	0.42–0.60	0.41–0.46
**15**	M. (Pa.) fansipanensis	30.9–44.3	41.7–42.5	1	1	1	0	0	0.53–0.80	0.49–0.59
**16**	M. (Pa.) hoanglienensis	37.4–47.6	59.6	1	1	1	0	0	0.54–0.75	0.44–0.63
**17**	M. (Pa.) huangshanensis	36.0–41.6	44.2	1	0	1	0	0	<0.50	0.42–0.45
**18**	M. (Pa.) insularis	36.8–41.2	47.1	1	1	1	0	1	0.46–0.57	0.40–0.43
**19**	M. (Pa.) jiangi	34.4–39.2	39.5–40.4	1	0	0	0	1	/	/
**20**	M. (Pa.) jingdongensis	53.0–56.5	63.5	1	1	1	2	2	/	0.58–0.59
**21**	M. (Pa.) jinggangensis	35.1–36.7	38.4–41.6	2	1	0	1	1	0.73–0.88	0.47–0.50
**22**	M. (Pa.) jiulianensis	30.4–33.9	34.1–37.5	1	1	1	0	1	0.50–0.59	0.44–0.48
**23**	M. (Pa.) kuatunensis	26.2–29.6	37.4	1	0	1	1	0	0.44	0.38–0.48
**24**	M. (Pa.) latidactyla	38.9	/	2	1	0	2	1	0.85	0.52
**25**	M. (Pa.) leishanensis	30.4–38.7	42.3	1	0	0	0	1	/	/
**26**	M. (Pa.) liboensis	60.5–67.7	60.8–70.6	2	1	1	2	1	0.48–0.78	0.44–0.61
**27**	M. (Pa.) lini	34.1–39.7	37.0–39.9	1	0	0	2	1	0.40–0.60	0.46–0.53
**28**	M. (Pa.) lishuiensis	30.7–34.7	36.9–40.4	1	0	0	0	0	/	/
**29**	M. (Pa.) minor	34.5–41.2	/	1	0	1	0	1	0.8–0.83	0.46–0.48
**30**	M. (Pa.) mufumontana	30.1–30.8	36.3	1	0	0	1	1	0.51–0.58	0.47–0.53
**31**	M. (Pa.) nankunensis	29.9–34.9	39.4–41.9	1	1	0	0	1	0.43–0.61	0.35–0.42
**32**	M. (Pa.) nanlingensis	30.5–37.3	/	1	1	1	1	1	0.43–0.57	0.45–0.51
**33**	M. (Pa.) obesa	35.6	37.5–41.2	1	0	0	0	1	0.51–0.66	0.41–0.47
**34**	M. (Pa.) ombrophila	27.4–34.5	32.8–35.0	1	0	0	0	0	0.53–0.69	0.33–0.41
**35**	M. (Pa.) omeimontis	56.0–59.5	68.0–72.5	1	1	1	1	1	/	0.52–0.56
**36**	M. (Pa.) palpebralespinosa	36.2–38.0	/	2	1	0	2	2	/	0.55
**37**	M. (Pa.) rubrimera	26.7–30.5	/	1	1	1	1	0	0.58–0.76	0.48–0.56
**38**	M. (Pa.) sangzhiensis	54.7	/	1	1	1	1	1	0.62	0.59
**39**	M. (Pa.) shuichengensis	102.0–118.3	99.8–115.6	2	0	1	2	2	0.67	0.43–0.47
**40**	M. (Pa.) shunhuangensis	30.3–33.7	37.6	1	0	0	0	1	0.40–0.63	0.50–0.55
**41**	M. (Pa.) spinata	47.2–54.4	54.0–55.0	1	0	1	2	2	0.43	0.56–0.58
**42**	M. (Pa.) tuberogranulatus	33.2–39.0	50.5	1	0	0	0	1	0.5	0.45–0.51
**43**	M. (Pa.) wugongensis	31.0–34.1	38.5–42.8	1	0	0	0	1	0.45–0.53	0.37–0.44
**44**	M. (Pa.) wuliangshanensis	27.3–31.6	41.3	1	0	1 or 0	0	0	0.5	0.50–0.51
**45**	M. (Pa.) wushanensis	30.4–35.5	38.4	1	0	0	0 (in female), 2 (in male)	1	0.5	0.47–0.48
**46**	M. (Pa.) xianjuensis	31.0–36.3	41.6	1	0	0	1	1	0.48–0.60	0.40–0.50

## Taxonomic accounts

### Megophrys (Panophrys) mirabilis

Taxon classificationAnimaliaAnuraMegophryidae

Lyu, Wang & Zhao sp. nov.

8EEA74E7-F477-5D9E-9511-762392E3F325

http://zoobank.org/E624C3F8-5522-4A3C-B376-3519B7E5A377

Figures 3 , 4A

#### Chresonymy.

*Megophrys* sp25 (SYS a002192–93, 2289, 2917 in Liu et al. 2018).

#### Type material.

**Figure 3.**
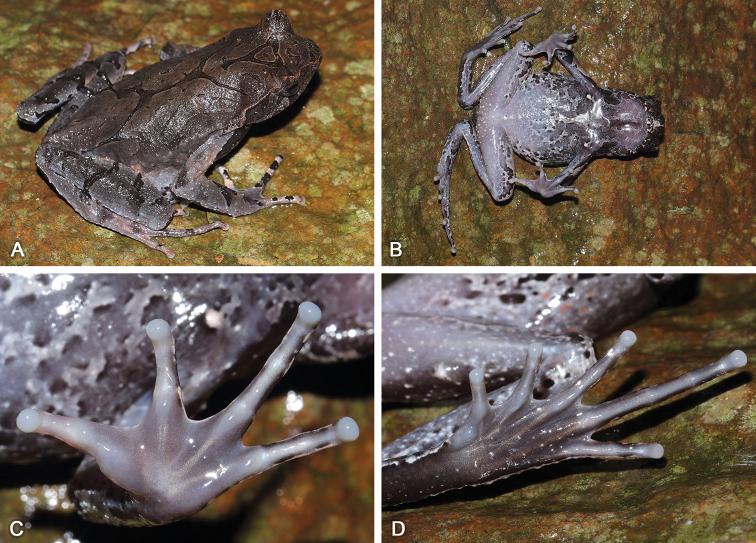
Adult male holotype SYS a002917 of Megophrys (Panophrys) mirabilis sp. nov. in life **A** dorsolateral view **B** ventral view **C** hand **D** foot.

**Figure 4.**
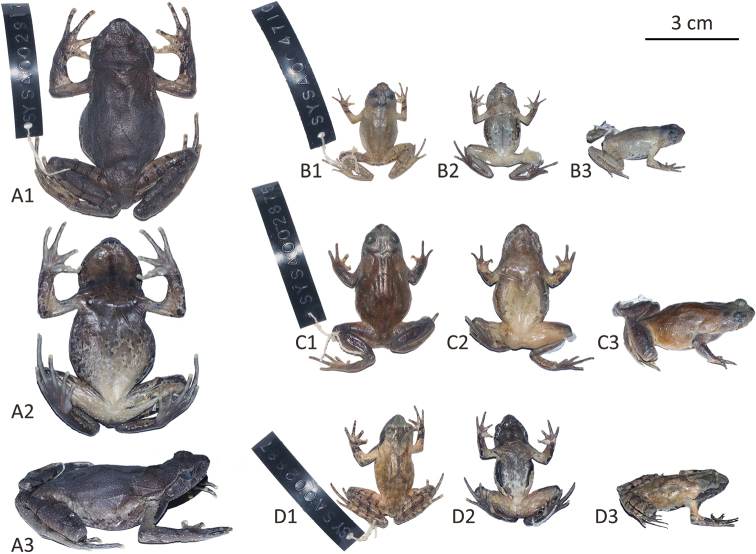
The holotype specimen of each new species in preservative **A**Megophrys (Panophrys) mirabilis sp. nov. **B**M. (Pa.) shimentaina sp. nov. **C**M. (Pa.) xiangnanensis sp. nov. **D**M. (Pa.) yangmingensis sp. nov. **1** dorsal view **2** ventral view **3** lateral view.

***Holotype*.**SYS a002917 (Figs 3, 4A), adult male, collected on 16 June 2014 by Yu-Long Li and Ying-Yong Wang from Huaping Nature Reserve (25.5554N, 109.9490E; ca 1300 m a.s.l.), Lingui District, Guilin City, Guangxi Zhuang Autonomous Region, PR China.

***Paratypes*.** Three adult specimens from the same locality as the holotype: male SYS a002192 and female SYS a002193 collected on 10 July 2013 by Jian Zhao and Yu-Long Li; female SYS a002289 collected on 9 September 2013 by Zu-Yao Liu.

#### Etymology.

The specific epithet *mirabilis* means marvelous, referring to its distinctive habitus and color pattern of this species within the subgenus Panophrys.

#### Common name.

Huaping Horned Toad (in English) / Huā Píng Jiăo Chán (花坪角蟾 in Chinese)

#### Diagnosis.

(1) Body size relatively large, SVL 55.8–61.4 mm (*N* = 2) in adult males and SVL 68.5–74.8 (*N* = 2) mm in adult females; (2) snout rounded in dorsal view; (3) internasal distance smaller than interorbital distance; (4) tympanum clear, moderate size, TD/ED 0.49–0.63; (5) absence of vomerine ridge and vomerine teeth; (6) tongue small, majorly attached to the mandible, free margin small and rounded, not notched behind; (7) hindlimbs slender, heels overlapping and tibio-tarsal articulation reaching forward at the central eye; (8) fingers with distinct lateral fringes, presence of indistinct subarticular tubercles at the bases; (9) toes with distinct lateral fringes and rudiment of webs, presence of indistinct subarticular tubercles at the bases; (10) presence of slightly large horn-like tubercle at the edge of upper eyelid; (11) dorsal skin smooth with granules, (12) skin on flanks flabby, with spiny tubercles; (13) supratympanic fold distinct, with dense tubercles, forming an extremely swollen large shoulder gland above insertion of arm; (14) grayish brown above, tinged with blue in males, but dorsum of head and body reddish brown in females; (15) ventral surface of throat and chest with grayish blue latticed patches and black spots in males, but with orange latticed patches and black spots in females; (16) presence of underdeveloped nuptial pads on the dorsal surface of the first finger in adult males.

#### Comparison.

Megophrys (Panophrys) mirabilis sp. nov. can be easily distinguished from all recognized congeners, by having a small tongue, majorly attached to the mandible, flank skin flabby with spiny tubercles, and supratympanic fold with dense tubercles forming an extremely swollen large shoulder gland above insertion of arm.

Further, detailed comparative data of Megophrys (Panophrys) mirabilis sp. nov. with 42 recognized congeners of *Panophrys* are given in Table 4.

Five *Panophrys* species were previously recorded from the hilly areas among Guangdong, Guangxi, and Hunan, namely Megophrys (Panophrys) acuta, M. (Pa.) brachykolos, M. (Pa.) nanlingensis, M. (Pa.) obesa, and M. (Pa.) shunhuangensis. M. (Pa.) mirabilis sp. nov. differs from M. (Pa.) acuta by the larger body size, SVL 55.8–61.4 mm in males and 68.5–74.8 mm in females (vs. 27.1–33.0 mm in males and 28.1–33.6 in females), snout rounded in dorsal view (vs. strongly remarkably pointed), fingers with distinct lateral fringes (vs. absent), and overlapping heels (vs. not meeting). M. (Pa.) mirabilis sp. nov. differs from M. (Pa.) brachykolos by the larger body size, SVL 55.8–61.4 mm in males and 68.5–74.8 mm in females (vs. 33.7–39.3 mm in males and 33.9–45.9 in females), slightly large horn-like tubercle at upper eyelid (vs. small), fingers and toes with distinct lateral fringes (vs. all absent), overlapping heels (vs. not meeting). M. (Pa.) mirabilis sp. nov. differs from M. (Pa.) nanlingensis by the larger body size, SVL 55.8–61.4 mm in males (vs. 30.5–37.3 mm), slightly large horn-like tubercle at upper eyelid (vs. small), absence of vomerine ridge and vomerine teeth (vs. both present), tongue not notched behind (vs. notched), and fingers with distinct lateral fringes (vs. absent). M. (Pa.) mirabilis sp. nov. differs from M. (Pa.) obesa by larger body size, SVL 55.8–61.4 mm in males and 68.5–74.8 mm in females (vs. 35.6 mm in male and 37.5–41.2 in females), slightly large horn-like tubercle at upper eyelid (vs. small), absence of vomerine ridge (vs. present), fingers and toes with distinct lateral fringes (vs. all absent), and overlapping heels (vs. not meeting). M. (Pa.) mirabilis sp. nov. differs from M. (Pa.) shunhuangensis by larger body size, SVL 55.8–61.4 mm in males and 68.5–74.8 mm in females (vs. 30.3–33.7 mm in males and 37.6 in female), slightly large horn-like tubercle at upper eyelid (vs. small), and fingers and toes with lateral fringes (vs. all absent).

With a large body size, SVL 55.8–61.4 mm in adult males and 68.5–74.8 mm in adult females, Megophrys (Panophrys) mirabilis sp. nov. is significantly different from 30 congeners whose SVL < 50 mm in males or < 60 mm in females, namely M. (Pa.) baolongensis, M. (Pa.) binchuanensis, M. (Pa.) boettgeri, M. (Pa.) cheni, M. (Pa.) daweimontis, M. (Pa.) dongguanensis, M. (Pa.) fansipanensis, M. (Pa.) hoanglienensis, M. (Pa.) huangshanensis, M. (Pa.) insularis, M. (Pa.) jiangi, M. (Pa.) jinggangensis, M. (Pa.) jiulianensis, M. (Pa.) kuatunensis, M. (Pa.) latidactyla, M. (Pa.) leishanensis, M. (Pa.) lini, M. (Pa.) lishuiensis, M. (Pa.) minor, M. (Pa.) mufumontana, M. (Pa.) nankunensis, M. (Pa.) ombrophila, M. (Pa.) palpebralespinosa, M. (Pa.) rubrimera, M. (Pa.) spinata, M. (Pa.) tuberogranulatus, M. (Pa.) wugongensis, M. (Pa.) wuliangshanensis, M. (Pa.) wushanensis, and M. (Pa.) xianjuensis.

Megophrys (Panophrys) mirabilis sp. nov. can be further distinguished from the remaining seven congeners by the following characteristics: SVL 55.8–61.4 mm in adult males and 68.5–74.8 mm in adult females [vs. SVL 45.1–51.0 mm in adult males in M. (Pa.) binlingensis; vs. SVL 81.3 mm in adult male in M. (Pa.) caudoprocta; vs. SVL 63.5 mm in adult female in M. (Pa.) jingdongensis; vs. SVL 102.0–118.3 mm in adult males and 99.8–115.6 mm in adult females in M. (Pa.) shuichengensis]; slightly large horn-like tubercle at upper eyelid [vs. small in M. (Pa.) binlingensis, M. (Pa.) jingdongensis, M. (Pa.) omeimontis, and M. (Pa.) sangzhiensis]; vomerine teeth absent [vs. present in M. (Pa.) caudoprocta, M. (Pa.) jingdongensis, M. (Pa.) liboensis, M. (Pa.) omeimontis, and M. (Pa.) sangzhiensis]; tongue not notched behind [vs. notched in M. (Pa.) binlingensis, M. (Pa.) jingdongensis, M. (Pa.) liboensis, M. (Pa.) omeimontis, M. (Pa.) sangzhiensis, and M. (Pa.) shuichengensis]; lateral fringes on toes narrow [vs. wide in M. (Pa.) jingdongensis, M. (Pa.) liboensis, and M. (Pa.) shuichengensis]; rudimentary webs on toes [vs. more than one-fourth webs in M. (Pa.) jingdongensis and M. (Pa.) shuichengensis].

#### Description of holotype.

Adult male. Body size large, SVL 61.4 mm; head width slightly larger than head length, HDW/HDL 1.02; snout rounded in dorsal view, projecting, sloping backward to mouth in profile, protruding well beyond margin of lower jaw; top of head flat; eyes large, ED 0.31 of HDL, pupil vertical; nostril oblique-ovoid; canthus rostralis well developed; loreal region slightly oblique; internasal distance smaller than interorbital distance; tympanum clear, TD/ED 0.49; large ovoid choanae at the base of the maxilla; absence of vomerine ridge and vomerine teeth; tongue small, majority attached at the mouth, margin rounded, not notched behind; absence of vocal sac.

Radio-ulna length 0.26 of SVL and hand 0.28 of SVL; hand without webs, fingers with distinct lateral fringes, relative finger length II < I < IV < III; tips of fingers slightly dilated, round; one indistinct subarticular tubercle at the bases of each finger; metacarpal tubercles indistinct, the inner one observably enlarged and the outer one smaller; presence of underdeveloped nuptial pad on the dorsal surface of the first finger, without nuptial spines. Hindlimbs slender, tibio-tarsal articulation reaching forward at the central eye when hindlimb is stretched along the side of the body; heels overlapping when the flexed hindlimbs are held at right angles to the body axis; tibia length 0.47 of SVL and foot length 0.71 of SVL; relative toe length I < II < V < III < IV; tips of toes round and slightly dilated; toes with narrow lateral fringes and rudiment of webs; one indistinct subarticular tubercle at the bases of each toe; inner metatarsal tubercle long ovoid and the outer one absent.

Dorsal skin smooth with sparse granules; flanks flabby with spiny tubercles; distinct supratympanic fold curving postero-ventrally from posterior corner of eye to a level above insertion of arm; small tubercles arranged from above the nostril, along the canthus rostralis, edge of upper eyelid and supratympanic fold, to the posterior margin of temporal region; a distinct horn-like prominent tubercle on the edge of upper eyelid; a discontinuous X-shaped ridge with several short ridges on two sides on the back; transverse skin ridges on the dorsal shank and thigh; ventral surface smooth; several tubercles on posterior hindlimbs; small pectoral gland closer to axilla; a single large femoral gland on rear of thigh.

#### Coloration.

Grayish brown above in life; an dark interorbital triangle with light colored center and edge; a dark X-shaped making with light edge on the central of dorsum; dark brown transverse bands on forearms and hindlimbs; supratympanic fold light gray; dark vertical band below the eye; iris grayish brown; ventral surface grayish white; throat and chest with grayish blue latticed patches and black spots; ventral hands and feet grayish white, tips of digits creamy white, metacarpal tubercle and metatarsal tubercle grayish white; pectoral gland and femoral gland white.

#### Variations.

Measurement data of type series are listed in Table 5. All paratypes are similar to the holotype. Females (SVL 68.5–74.8 mm) are significantly larger than males (SVL 55.8–61.4 mm). Dorsal surfaces reddish brown and ventral surfaces with orange latticed patches and black spots in females SYS a002193, 2289.

#### Distribution and ecology.

Currently, Megophrys (Panophrys) mirabilis sp. nov. is only known from Huaping Nature Reserve, northeastern Guangxi. The individuals were found on shrubbery branches near trail paths between elevations of 1300–1330 m a.s.l. from June to September. Males were not calling when found, but the collected female specimens bear mature yellowish oocytes. Tadpoles have not been found and ecological information remains unknown.

**Table 5.** Measurements (in mm) of the type series of Megophrys (Panophrys) mirabilis sp. nov., * for the holotype.

	SYS a002917 *	SYS a002192	SYS a002193	SYS a002289
**Sex**	Male	Male	Female	Female
**SVL**	61.4	55.8	74.8	68.5
**HDL**	21.4	18.8	23.7	22.6
**HDW**	21.8	18.8	23.9	22.4
**SNT**	7.8	7.1	9.0	8.8
**IND**	6.7	5.9	7.5	6.8
**IOD**	7.2	6.5	8.1	7.6
**ED**	6.7	5.9	8.1	6.8
**TD**	3.3	3.2	4.3	4.3
**TED**	3.3	3.2	4.2	3.7
**HND**	17.3	15.3	20.2	19.5
**RAD**	15.9	13.9	18.3	17.6
**FTL**	43.7	37.8	48.8	43.2
**TIB**	28.9	26.3	33.8	30.5

### Megophrys (Panophrys) shimentaina

Taxon classificationAnimaliaAnuraMegophryidae

Lyu, Liu & Wang sp. nov.

16A2525B-4362-51D6-95BB-236A8B03FFB9

http://zoobank.org/E9F8A869-8923-4C0F-8750-181EE0843A07

Figures 4B , 5 , 6A

#### Chresonymy.

*Megophrys* sp29 (SYS a002077, 2081, 4172–4173 in Liu et al. 2018)

#### Type material.

**Figure 5.**
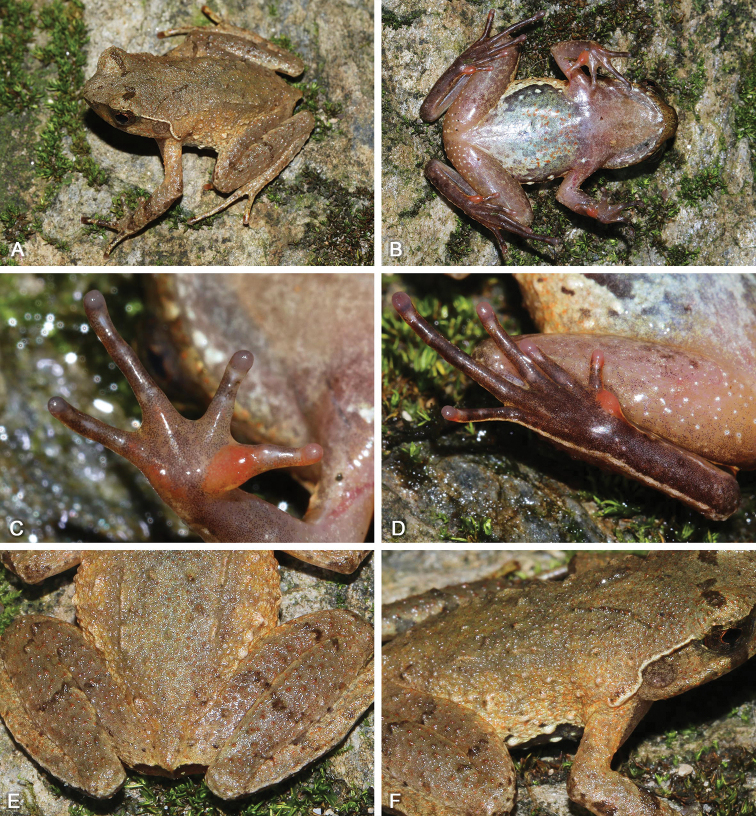
Adult male holotype SYS a004710 of Megophrys (Panophrys) shimentaina sp. nov. in life **A** dorsolateral view **B** ventral view **C** hand **D** foot **E** posterior view **F** large warts on the flanks and absence of conical spines on supratympanic fold.

***Holotype*.**SYS a004710 (Figs 4B, 5), adult male, collected on 27 April 2016 by Zhi-Tong Lyu and Yuan-Qiu Li from Shimentai Nature Reserve (24.4095N, 113.1095E; ca 370 m a.s.l.), Yingde City, Qingyuan City, Guangdong Province, PR China.

***Paratypes*.** Eleven adult males from the same locality as the holotype: SYS a002077, 2081–2085, collected on 25–26 April 2013 by Run-Lin Li and Yuan-Qiu Li; SYS a004172–4173, collected on 27 July 2015 by Ying-Yong Wang and Yuan-Qiu Li; SYS a005448/CIB 110015 collected on 19 August 2016 and SYS a005992–5993 collected on 20 June 2017 by Zhi-Tong Lyu and Yong-You Zhao.

#### Etymology.

The specific epithet *shimentaina* refers to its type locality, Shimentai Nature Reserve.

#### Common name.

Shimentai Horned Toad (in English) / Shí Mén Taí Jiăo Chán (石门台角蟾in Chinese)

#### Diagnosis.

(1) Body size small, SVL 28.0–30.6 (28.9 ± 0.9, *N* = 12) mm in adult males; (2) snout rounded in dorsal view; (3) tympanum clear, TD/ED 0.57–0.66; (4) presence of weak vomerine ridge and vomerine teeth; (5) margin of tongue rounded, not notched behind; (6) hindlimbs slender, heels overlapping and tibio-tarsal articulation reaching forward between tympanum to anterior corner of eye; (7) tibia 0.44–0.53 of SVL and foot 0.62–0.76 of SVL; (8) fingers with narrow lateral fringes, presence of indistinct subarticular tubercles at the bases; (9) toes with narrow lateral fringes and rudiment of webs, absence of subarticular tubercle; (10) presence of a small horn-like tubercle at the edge of upper eyelid; (11) presence of tiny, barely visible, black to dark brown spines on the whole dorsal skin, flanks, dorsal limbs, the region around cloaca, and rear of hindlimbs; (12) dorsal skin rough, a discontinuous “/ \”-shaped ridge with two discontinuous dorsolateral ridges on two sides on the back; (13) several large warts on the flanks; (14) supratympanic fold distinct and white, with tiny spines; (15) light brown above, a dark brown stripe on each upper eyelid; (16) single subgular vocal sac in males; (17) weak nuptial pads with serried olive nuptial spines, on the dorsal surface of the first and second fingers in adult males.

#### Comparison.

Comparative data of Megophrys (Panophrys) shimentaina sp. nov. with M. (Pa.) mirabilis sp. nov. and 42 recognized congeners of *Panophrys* are given in Table 4.

Megophrys (Panophrys) shimentaina sp. nov. differs from M. (Pa.) mirabilis sp. nov. by the smaller body size, SVL 28.0–30.6 mm in males (vs. 55.8–61.4 mm in males), small horn-like tubercle at upper eyelid (vs. slightly large), presence of vomerine teeth (vs. absent), the presence of tiny spines on the whole dorsal skin, flanks, dorsal limbs, the region around cloaca, and rear of hindlimbs (vs. such spines absent), presence of vocal sac in males (vs. absent), and presence of nuptial spines in males (vs. absent).

Compared with the five *Panophrys* species previously recorded from the hilly areas among Guangdong, Guangxi, and Hunan, Megophrys (Panophrys) shimentaina sp. nov. differs from M. (Pa.) acuta by the small horn-like tubercle at upper eyelid (vs. slightly large), snout rounded in dorsal view (vs. strongly remarkably pointed), presence of vomerine teeth (vs. absent), presence of tiny spines on the whole dorsal skin, flanks, dorsal limbs, the region around cloaca, and rear of hindlimbs (vs. such spines absent), and overlapping heels (vs. not meeting). M. (Pa.) shimentaina sp. nov. differs from M. (Pa.) brachykolos by the smaller body size SVL 28.0–30.6 mm in males (vs. 33.7–39.3 mm in males), presence of vomerine teeth (vs. absent), presence of tiny spines on the whole dorsal skin, flanks, dorsal limbs, the region around cloaca, and rear of hindlimbs (vs. such spines absent), narrow lateral fringes on toes (vs. absent), and overlapping heels (vs. not meeting). M. (Pa.) shimentaina sp. nov. differs from M. (Pa.) nanlingensis by the presence of tiny spines on the whole dorsal skin, flanks, dorsal limbs, the region around cloaca, and rear of hindlimbs (vs. such spines absent), and tongue not notched behind (vs. notched). M. (Pa.) shimentaina sp. nov. differs from M. (Pa.) obesa by the smaller body size SVL 28.0–30.6 mm in males (vs. 35.6 mm in single male), presence of vomerine teeth (vs. absent), presence of tiny spines on the whole dorsal skin, flanks, dorsal limbs, the region around cloaca, and rear of hindlimbs (vs. such spines absent), narrow lateral fringes on toes (vs. absent), and overlapping heels (vs. not meeting). M. (Pa.) shimentaina sp. nov. differs from M. (Pa.) shunhuangensis by the presence of vomerine teeth (vs. absent), tibio-tarsal articulation reaching forward between tympanum to anterior corner of eye (vs. at the eye), and the presence of tiny spines on the whole dorsal skin, flanks, dorsal limbs, the region around cloaca, and rear of hindlimbs (vs. such spines absent).

With a small body size, SVL 28.0–30.6 mm in adult males, Megophrys (Panophrys) shimentaina sp. nov. is significantly different from 15 congeners whose SVL > 35 mm in males, namely M. (Pa.) baolongensis, M. (Pa.) binlingensis, M. (Pa.) caudoprocta, M. (Pa.) hoanglienensis, M. (Pa.) huangshanensis, M. (Pa.) insularis, M. (Pa.) jingdongensis, M. (Pa.) jinggangensis, M. (Pa.) latidactyla, M. (Pa.) liboensis, M. (Pa.) omeimontis, M. (Pa.) palpebralespinosa, M. (Pa.) sangzhiensis, M. (Pa.) shuichengensis, and M. (Pa.) spinata.

Megophrys (Panophrys) shimentaina sp. nov. can be further distinguished from the remaining 22 congeners by the following characteristics: vomerine teeth present [vs. absent in M. (Pa.) binchuanensis, M. (Pa.) boettgeri, M. (Pa.) cheni, M. (Pa.) jiangi, M. (Pa.) kuatunensis, M. (Pa.) leishanensis, M. (Pa.) lini, M. (Pa.) lishuiensis, M. (Pa.) minor, M. (Pa.) mufumontana, M. (Pa.) ombrophila, M. (Pa.) tuberogranulatus, M. (Pa.) wugongensis, M. (Pa.) wuliangshanensis, M. (Pa.) wushanensis, and M. (Pa.) xianjuensis]; tongue not notched behind [vs. notched in M. (Pa.) cheni, M. (Pa.) boettgeri, M. (Pa.) fansipanensis, M. (Pa.) jiulianensis, M. (Pa.) kuatunensis, M. (Pa.) minor, and M. (Pa.) rubrimera]; lateral fringes on toes narrow [vs. absent in M. (Pa.) daweimontis, M. (Pa.) dongguanensis, M. (Pa.) fansipanensis, M. (Pa.) jiangi, M. (Pa.) jiulianensis, M. (Pa.) leishanensis, M. (Pa.) lishuiensis, M. (Pa.) minor, M. (Pa.) nankunensis, M. (Pa.) ombrophila, M. (Pa.) tuberogranulatus, M. (Pa.) wugongensis, and M. (Pa.) wuliangshanensis; wide in M. (Pa.) binchuanensis, M. (Pa.) boettgeri, M. (Pa.) cheni, and M. (Pa.) lini; vs. absent in females while wide in males in M. (Pa.) wushanensis]; rudimentary webs on toes [vs. lacking webs in M. (Pa.) daweimontis, M. (Pa.) fansipanensis, M. (Pa.) kuatunensis, M. (Pa.) lishuiensis, M. (Pa.) ombrophila, M. (Pa.) rubrimera, and M. (Pa.) wuliangshanensis].

**Figure 6.**
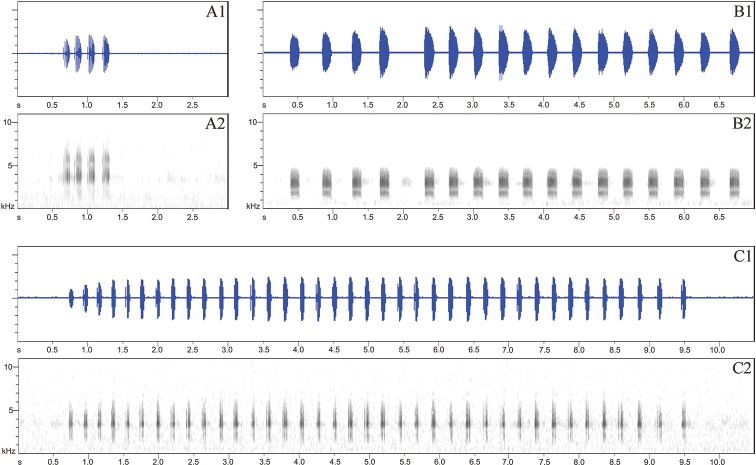
Advertisement calls spectrograms **A**Megophrys (Panophrys) shimentaina sp. nov. **B**M. (Pa.) xiangnanensis sp. nov. **C**M. (Pa.) yangmingensis sp. nov. **1** sonogram **2** waveform.

#### Description of holotype.

Adult male. Body size small, SVL 28.4 mm; head width slightly smaller than head length, HDW/HDL 0.95; snout rounded in dorsal view, projecting, sloping backward to mouth in profile, protruding well beyond margin of lower jaw; top of head flat; eyes large, ED 0.33 of HDL, pupil vertical; nostril oblique-ovoid; canthus rostralis well developed; loreal region slightly oblique; internasal distance slightly larger than interorbital distance; tympanum clear, in medium size, TD/ED 0.61; large ovoid choanae at the base of the maxilla; presence of weak vomerine ridge and vomerine teeth; margin of tongue rounded, not notched behind; presence of a single subgular vocal sac, a pair of slit-like openings at posterior of jaw.

Radio-ulna length 0.22 of SVL and hand 0.26 of SVL; hand without webs, fingers with narrow lateral fringes, relative finger length I ≈ II < IV < III; tips of fingers slightly dilated, round; one indistinct subarticular tubercle at the bases of each finger; inner metacarpal tubercle observably enlarged and the outer one smaller; nuptial pads with serried olive nuptial spines on the dorsal surface of the first and second fingers. Hindlimbs slender, tibio-tarsal articulation reaching forward to the posterior corner of eye when hindlimb is stretched along the side of the body; heels overlapping when the flexed hindlimbs are held at right angles to the body axis; tibia length 0.47 of SVL and foot length 0.67 of SVL; relative toe length I < II < V < III < IV; tips of toes round and slightly dilated; toes with distinct lateral fringes and rudiment of webs, without subarticular tubercle; inner metatarsal tubercle long ovoid and the outer one absent.

Dorsal skin rough; numerous granules densely arranged on the top of head, loreal region, lips, temporal region, dorsal body, flanks and dorsal limbs; several tubercles on upper eyelid, including a horn-like prominent tubercle on the edge; all granules and tubercles bearing tiny, barely visible spines; clear supratympanic fold with tiny spines, curving postero-ventrally from posterior corner of eye to a level above insertion of arm; tubercles and granules forming discontinuous “/ \”-shaped ridge and two discontinuous dorsolateral ridges on two sides at the central back; large tubercles and warts on the flanks; ventral surface smooth; several granules bearing black spines on the region around cloaca and rear of hindlimbs; small pectoral gland closer to axilla; a single large femoral gland on rear of thigh.

#### Coloration.

Light brown above in life; a dark brown stripe on dorsal surface of each eye; narrow dark brown transverse bands on forearms and hindlimbs; supratympanic fold white; dark vertical band below the eye; iris reddish brown; all spines black or dark brown; ventral surface pale; throat flesh color; scarlet spots on the chest; a large white blotch on the belly; a pair of lateroventral longitudinal broad black stripes with several white tubercles on two sides; ventral limbs flesh color with white spots; ventral hands and ventral feet brown, tips of digits pale brown; metacarpal tubercle and metatarsal tubercle reddish; pectoral gland and femoral gland white.

#### Variations.

Measurement data of type series are listed in Table 6. All paratypes are extremely similar to the holotype but SYS a002082 has an “X” pattern on its back.

#### Distribution and ecology.

Currently, Megophrys (Panophrys) shimentaina sp. nov. is known only from Shimentai Nature Reserve, northern Guangdong. This toad is uncommon in its distribution areas. All individuals were found from two slowly flowing mountain streams between elevations of 210–500 m a.s.l. Males call on plant leaves from April to August, suggesting their breeding season corresponds to this period. Females and tadpoles have not been found.

#### Vocalization.

The advertisement calls of Megophrys (Panophrys) shimentaina sp. nov. were recorded from four males at 18–20 °C air temperature on 27 April 2016. Thirty calls with 96 notes are measured and the spectrograms are shown in Fig. 6A. The advertisement call is made up of 3.8 ± 0.4 (3–4, *N* = 30) continuous click notes. Each call lasts 0.50 ± 0.07 s (0.36–0.58 s, *N* = 30) and each note lasts 85 ± 8 ms (64–101 ms, *N* = 96) with an interval of 67 ± 14 ms (44–121 ms, *N* = 71) between every two notes. The peak frequency measures at 4895 ± 124 Hz (4688–5156 Hz, *N* = 96).

**Table 6.** Measurements (in mm) of the type series of Megophrys (Panophrys) shimentaina sp. nov., * for the holotype.

	SYS a004710*	**SYS a002077**	**SYS a002081**	**SYS a002082**	**SYS a002083**	**SYS a002084**	**SYS a002085**	**SYS a004172**	**SYS a004173**	**SYS a005448 / CIB 110015**	**SYS a005992**	**SYS a005993**
**Sex**	Male	Male	Male	Male	Male	Male	Male	Male	Male	Male	Male	Male
**SVL**	28.4	28.5	28.1	30.6	29.0	29.2	28.8	28.0	30.4	28.0	29.3	28.7
**HDL**	10.0	10.1	9.9	10.4	10.1	10.1	10.0	9.9	10.5	10.7	10.1	10.1
**HDW**	9.6	9.5	9.5	10.0	9.6	9.7	9.5	9.8	10.0	10.3	9.8	9.9
**SNT**	3.3	3.4	3.3	3.5	3.3	3.4	3.4	3.3	3.4	3.2	3.4	3.4
**IND**	3.0	3.0	3.0	3.1	3.1	3.1	3.0	3.1	3.2	2.8	3.1	3.0
**IOD**	2.6	2.8	2.8	2.9	2.8	2.8	2.7	2.8	2.9	3.1	2.8	2.6
**ED**	3.3	3.2	3.3	3.4	3.3	3.3	3.3	3.4	3.4	3.4	3.4	3.4
**TD**	2.0	1.8	1.9	2.2	2.0	1.9	2.0	1.9	2.1	2.1	2.2	2.1
**TED**	1.6	1.6	1.5	1.6	1.7	1.5	1.5	1.4	1.6	1.5	1.5	1.5
**HND**	7.4	7.2	7.3	7.5	7.5	7.4	7.3	6.8	7.2	7.8	7.1	7.5
**RAD**	6.2	6.1	6.2	6.4	6.2	6.2	6.0	5.5	6.1	6.3	6.0	6.4
**FTL**	19.1	19.9	19.0	20.5	19.4	19.3	19.3	17.9	18.7	21.4	19.4	20.3
**TIB**	13.5	14.2	13.2	14.9	13.4	14.3	13.6	12.8	13.3	14.9	13.4	14.6

### Megophrys (Panophrys) xiangnanensis

Taxon classificationAnimaliaAnuraMegophryidae

Lyu, Zeng & Wang sp. nov.

E8FD5215-EBFB-55AF-A138-6FA0F693AF26

http://zoobank.org/F27079DE-C1AF-4B00-900F-1E1783C58762

Figures 4C , 6B , 7

#### Chresonymy.

*Megophrys* sp2 (SYS a002874–76, 2878–79 in Liu et al. 2018)

#### Holotype.

**Figure 7.**
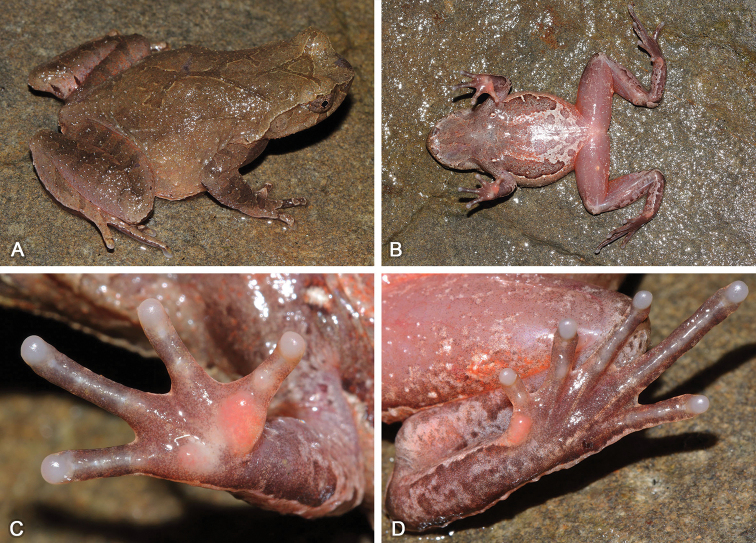
Adult male holotype SYS a002875 of Megophrys (Panophrys) xiangnanensis sp. nov. in life **A** dorsolateral view **B** ventral view **C** hand **D** foot.

SYS a002875 (Figs 4C, 7), adult male, collected on 12 June 2014 by Yu-Long Li and Ying-Yong Wang from Mt Yangming (26.1177N, 111.8945E; ca 1360 m a.s.l.), Shuangpai County, Yongzhou City, Hunan Province, PR China.

#### Paratypes.

Eleven adult specimens, female SYS a002874 and males SYS a002876/CIB 116072 and SYS a002878–2886, collected at the same time from the same locality as the holotype.

#### Etymology.

The specific epithet *xiangnanensis* is an adjective derived from Chinese Pinyin Xiāng Nán, which means southern Hunan, for the distribution area of this species.

#### Common name.

Southern Hunan Horned Toad (in English) / Xiāng Nán Jiăo Chán (湘南角蟾 in Chinese)

#### Diagnosis.

(1) Moderate body size, SVL 38.6–42.0 mm (40.3 ± 1.3, *N* = 11) in adult males and SVL 44.4 mm in adult female; (2) snout rounded in dorsal view; (3) tympanum clear, TD/ED 0.38–0.49; (4) presence of weak vomerine ridge, absence of vomerine teeth; (5) margin of tongue rounded, not notched behind; (6) hindlimbs slender, heels just meeting and tibio-tarsal articulation reaching forward between eye and tympanum; (7) tibia 0.41–0.46 of SVL and foot 0.57–0.62 of SVL; (8) fingers without lateral fringes, presence of distinct subarticular tubercles at the bases; (9) toes with relatively wide lateral fringes and rudiment of webs, presence of distinct subarticular tubercles at the bases; (10) presence of small horn-like tubercle at the edge of upper eyelid; (11) dorsal skin smooth with sparse granules, a discontinuous X-shaped ridge with two discontinuous dorsolateral ridges on two side on the back; (12) sparse tubercles on the flanks; (13) supratympanic fold light colored; (14) single subgular vocal sac in males; (15) presence of nuptial pads on the dorsal surface of the first and second fingers in adult males.

#### Comparison.

Comparative data of Megophrys (Panophrys) xiangnanensis sp. nov. with M. (Pa.) mirabilis sp. nov., M. (Pa.) shimentaina sp. nov., and 42 recognized congeners of *Panophrys* are given in Table 4.

Megophrys (Panophrys) xiangnanensis sp. nov. differs from M. (Pa.) mirabilis sp. nov. by the smaller body size, SVL 38.6–42.0 mm in males and 44.4 mm in single female (vs. 55.8–61.4 mm in males and 68.5–74.8 in females), small horn-like tubercle at upper eyelid (vs. slightly large), wide lateral fringes on toes (vs. narrow), heels just meeting (vs. overlapping), presence of vocal sac in males (vs. absent), and presence of nuptial spines in males (vs. absent).

Megophrys (Panophrys) xiangnanensis sp. nov. differs from M. (Pa.) shimentaina sp. nov. by the larger body size, SVL 38.6–42.0 mm in males (vs. 28.0–30.6 mm in males), absence of vomerine teeth (vs. present), wide lateral fringes on toes (vs. narrow), and heels just meeting (vs. overlapping).

Compared with the five *Panophrys* species previously recorded from the hilly areas among Guangdong, Guangxi and Hunan, Megophrys (Panophrys) xiangnanensis sp. nov. differs from M. (Pa.) acuta by the larger body size, SVL 38.6–42.0 mm in males and 44.4 mm in single female (vs. 27.1–33.0 mm in males and 28.1–33.6 mm in females), small horn-like tubercle at upper eyelid (vs. slightly large), snout rounded in dorsal view (vs. strongly remarkably pointed), wide lateral fringes on toes (vs. narrow), and heels just meeting (vs. not meeting). M. (Pa.) xiangnanensis sp. nov. differs from M. (Pa.) brachykolos by the wide lateral fringes on toes (vs. absent), and heels just meeting (vs. not meeting). M. (Pa.) xiangnanensis sp. nov. differs from M. (Pa.) nanlingensis by the larger body size, SVL 38.6–42.0 mm in males (vs. 30.5–37.3 mm in males), absence of vomerine teeth (vs. present), tongue not notched behind (vs. notched), wide lateral fringes on toes (vs. narrow) , and heels just meeting (vs. overlapping). M. (Pa.) xiangnanensis sp. nov. differs from M. (Pa.) obesa by the larger body size, SVL 38.6–42.0 mm in males and 44.4 mm in single female (vs. 35.6 mm in single male and 37.5–41.2 mm in females), wide lateral fringes on toes (vs. absent), and heels just meeting (vs. not meeting). M. (Pa.) xiangnanensis sp. nov. differs from M. (Pa.) shunhuangensis by the larger body size, SVL 38.6–42.0 mm in males and 44.4 mm in single female (vs. 30.3–33.7 mm in males and 37.6 in female), wide lateral fringes on toes (vs. absent), and heels just meeting (vs. overlapping).

With a moderate body size, SVL 38.6–42.0 mm in adult males, Megophrys (Panophrys) xiangnanensis sp. nov. is significantly different from 18 congeners whose SVL< 35 mm or > 45 mm in males, namely M. (Pa.) binlingensis, M. (Pa.) caudoprocta, M. (Pa.) cheni, M. (Pa.) jingdongensis, M. (Pa.) jiulianensis, M. (Pa.) kuatunensis, M. (Pa.) liboensis, M. (Pa.) lishuiensis, M. (Pa.) mufumontana, M. (Pa.) nankunensis, M. (Pa.) ombrophila, M. (Pa.) omeimontis, M. (Pa.) rubrimera, M. (Pa.) sangzhiensis, M. (Pa.) shuichengensis, M. (Pa.) spinata, M. (Pa.) wugongensis, and M. (Pa.) wuliangshanensis.

Megophrys (Panophrys) xiangnanensis sp. nov. can be further distinguished from the remaining 19 congeners by the following characteristics: small horn-like tubercle at upper eyelid [vs. slightly large in M. (Pa.) jinggangensis, M. (Pa.) latidactyla, and M. (Pa.) palpebralespinosa]; vomerine teeth absent [vs. present in M. (Pa.) daweimontis, M. (Pa.) dongguanensis, M. (Pa.) fansipanensis, M. (Pa.) hoanglienensis, M. (Pa.) insularis, M. (Pa.) jinggangensis, M. (Pa.) latidactyla, and M. (Pa.) palpebralespinosa]; tongue not notched behind [vs. notched in M. (Pa.) baolongensis, M. (Pa.) boettgeri, M. (Pa.) fansipanensis, M. (Pa.) hoanglienensis, M. (Pa.) huangshanensis, M. (Pa.) insularis, and M. (Pa.) minor]; lateral fringes on toes wide [vs. absent in M. (Pa.) baolongensis, M. (Pa.) daweimontis, M. (Pa.) dongguanensis, M. (Pa.) fansipanensis, M. (Pa.) hoanglienensis, M. (Pa.) huangshanensis, M. (Pa.) insularis, M. (Pa.) jiangi, M. (Pa.) leishanensis, M. (Pa.) minor, and M. (Pa.) tuberogranulatu; vs. narrow in M. (Pa.) jinggangensis and M. (Pa.) xianjuensis; vs. absent in females while wide in males in M. (Pa.) wushanensis]; rudimentary webs on toes [vs. more than one-fourth webs in M. (Pa.) palpebralespinosa; vs. lacking webs in M. (Pa.) baolongensis, M. (Pa.) daweimontis, M. (Pa.) fansipanensis, M. (Pa.) hoanglienensis, and M. (Pa.) huangshanensis].

#### Description of holotype.

Adult male. Moderate body size, SVL 40.9 mm; head width slightly larger than head length, HDW/HDL 1.02; snout rounded in dorsal view, projecting, sloping backward to mouth in profile, protruding well beyond margin of lower jaw; top of head flat; eyes large, ED 0.41 of HDL, pupil vertical; nostril oblique-ovoid; canthus rostralis well developed; loreal region slightly oblique; internasal distance slightly larger than interorbital distance; tympanum clear, TD/ED 0.44; large ovoid choanae at the base of the maxilla; presence of weak vomerine ridge, absence of vomerine teeth; margin of tongue rounded, not notched behind; presence of a single subgular vocal sac, a pair of slit-like openings at posterior of jaw.

Radio-ulna length 0.22 of SVL and hand 0.23 of SVL; hand without webs, fingers without lateral fringes, relative finger length I < II < IV < III; tips of fingers slightly dilated, round; one distinct subarticular tubercle at the bases of each finger; inner metacarpal tubercle observably enlarged and the outer one smaller; a single nuptial pad on the dorsal surface of the first and second fingers. Hindlimbs slender, tibio-tarsal articulation reaching forward between eye and tympanum when hindlimb is stretched along the side of the body; heels just meeting when the flexed hindlimbs are held at right angles to the body axis; tibia length 0.42 of SVL and foot length 0.58 of SVL; relative toe length I < II < V < III < IV; tips of toes round and slightly dilated; toes with relatively wide lateral fringes and rudiment of webs; one distinct subarticular tubercle at the bases of each toe; inner metatarsal tubercle long ovoid and the outer one absent.

Dorsal skin smooth with sparse granules; sparse tubercles on the flanks; a horn-like prominent tubercle on the edge; clear supratympanic fold curving postero-ventrally from posterior corner of eye to a level above insertion of arm; a discontinuous X-shaped ridge and two discontinuous dorsolateral ridges on two sides at the central back; sparse tubercles on the dorsal shank and thigh; ventral surface smooth; several tubercles on posterior hindlimbs; small pectoral gland closer to axilla; a single large femoral gland on rear of thigh.

#### Coloration.

Yellowish brown above in life; a dark interorbital triangle with light colored center and edge; a dark X-shaped making with light edge on the central of dorsum; dark brown transverse bands on forearms and hindlimbs; supratympanic fold light colored; dark vertical band below the eye; iris light brown with net-like stripes; throat and anterior chest reddish gray; a longitudinal stripe on the throat; a large white blotch with scarlet spots on the belly; one pair of lateroventral longitudinal broad reddish stripes on two sides; ventral limbs flesh color; ventral hands purplish, tips of fingers pale-grey, metacarpal tubercle reddish; ventral feet purplish brown, tips of fingers pale grey, metatarsal tubercle reddish; pectoral gland and femoral gland white.

#### Variations.

Measurement data of type series are listed in Table 7. All paratypes are similar to the holotype. Female (SVL 44.4 mm) are slightly larger than males (SVL 38.6–42.0 mm).

#### Distribution and ecology.

Megophrys (Panophrys) xiangnanensis sp. nov. is currently known only from Mt Yangming, southwestern Hunan. This toad inhabits areas near slowly flowing mountain streams surrounded by moist subtropical secondary evergreen broadleaf forests between elevations of 900–1400 m a.s.l. Males call from May to July, and during this time the males bear nuptial pads. Only one female individual was found, and tadpoles and other ecological information remain unknown.

#### Vocalization.

The advertisement calls of Megophrys (Panophrys) xiangnanensis sp. nov. were recorded from the Holotype at 16 °C air temperature on 12 June 2014. Four calls with 98 notes are measured and the spectrograms are shown in Fig. 6B. The advertisement call is made up of 24.5 ± 4.7 (17–29, *N* = 4) continuous click notes. Each call lasts 9.46 ± 1.77 s (6.39–10.53 s, *N* = 4) and each note lasts 151 ± 12 ms (113–177 ms, *N* = 98) with an interval of 240 ± 95 ms (148–631 ms, *N* = 94) between every two notes. The peak frequency measures at 3033 ± 123 Hz (2813–3188 Hz, *N* = 98).

**Table 7.** Measurements (in mm) of the type series of Megophrys (Panophrys) xiangnanensis sp. nov., * for the holotype.

	SYS a002875*	**SYS a002876 / CIB 116072**	**SYS a002878**	**SYS a002879**	**SYS a002880**	**SYS a002881**	**SYS a002882**	**SYS a002883**	**SYS a002884**	**SYS a002885**	**SYS a002886**	**SYS a002874**
**Sex**	Male	Male	Male	Male	Male	Male	Male	Male	Male	Male	Male	Female
**SVL**	40.9	38.7	39.0	40.2	38.6	40.5	41.7	41.5	42.0	41.0	39.1	44.4
**HDL**	13.2	12.6	12.3	13.1	12.9	13.0	13.1	13.2	13.4	13.0	12.8	14.0
**HDW**	13.5	12.5	12.6	13.3	12.8	13.2	13.1	13.2	14.0	13.1	13.0	14.3
**SNT**	4.5	4.4	4.3	4.4	4.3	4.6	4.2	4.5	4.7	4.6	4.3	5.0
**IND**	4.5	4.2	4.2	4.5	4.3	4.0	4.4	4.6	4.5	4.5	4.3	4.5
**IOD**	3.7	3.6	4.0	4.0	3.8	3.7	3.7	4.0	3.8	3.9	3.8	44.3
**ED**	5.4	4.6	5.0	5.1	4.8	5.0	5.0	5.1	5.1	5.0	5.1	5.5
**TD**	2.4	2.1	2.0	2.4	2.1	1.9	2.2	2.1	2.5	2.2	2.2	2.7
**TED**	2.3	2.4	2.0	2.3	2.1	2.9	2.3	2.5	2.1	2.5	2.2	2.4
**HND**	9.3	9.0	8.8	9.0	8.9	10.3	9.3	8.9	9.0	9.3	9.2	9.8
**RAD**	9.0	8.7	8.8	8.8	8.9	9.8	9.3	8.9	9.0	9.3	9.2	9.8
**FTL**	23.9	23.0	24.3	22.9	23.1	24.8	24.2	24.3	24.3	23.5	23.8	27.6
**TIB**	17.0	17.9	17.8	17.0	17.9	18.2	18.2	18.2	17.3	17.4	17.7	19.1

### Megophrys (Panophrys) yangmingensis

Taxon classificationAnimaliaAnuraMegophryidae

Lyu, Zeng & Wang sp. nov.

0D94F88F-2455-5F77-8592-1AC34EF7D6E8

http://zoobank.org/D466B824-AE2D-4EAA-94D1-A6BF39534942

Figures 4D , 6C , 8

#### Chresonymy.

*Megophrys* sp28 (SYS a002877, 2888–2890 in Liu et al. 2018)

#### Holotype.

**Figure 8.**
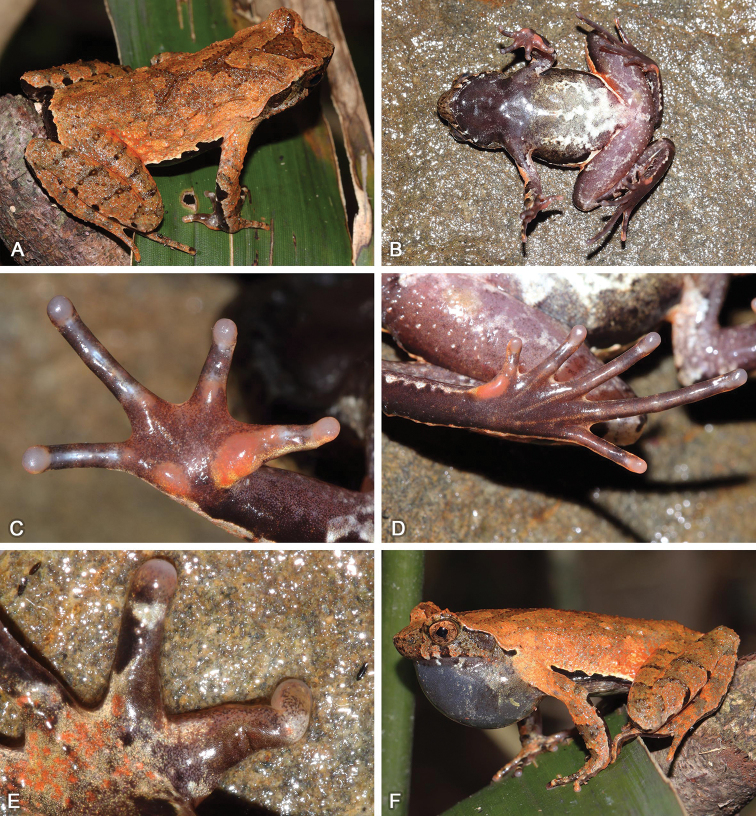
Adult male holotype SYS a002887 of Megophrys (Panophrys) yangmingensis sp. nov. in life **A** dorsolateral view **B** ventral view **C** hand **D** foot **E** villiform black nuptial spines **F** single subgular vocal sac.

SYS a002887 (Figs 4D, 8), adult male, collected on 12 June 2014 by Yu-Long Li and Ying-Yong Wang from Mt Yangming (26.1177N, 111.8945E; ca 1360 m a.s.l.), Shuangpai County, Yongzhou City, Hunan Province, PR China.

#### Paratypes.

Seven adult specimens from the same locality as the holotype: female SYS a002877, and males SYS a2888–2889, 2891–2892, collected at the same time as the holotype; male SYS a002307 and SYS a002310/CIB 116073, collected on 8 September 2013 by Zu-Yao Liu.

#### Etymology.

The specific epithet *yangmingensis* refers to its type locality, Mt Yangming.

#### Common name.

Mt Yangming Horned Toad (in English) / Yáng Míng Shān Jiăo Chán (阳明山角蟾in Chinese)

#### Diagnosis.

(1) Body size small, SVL 33.2–37.1 mm (35.3 ± 1.4, *N* = 7) in adult males and SVL 45.2 mm in adult female; (2) snout rounded in dorsal view; (3) tympanum clear, TD/ED 0.42–0.50; (4) presence of weak vomerine ridge, absence of vomerine teeth; (5) margin of tongue rounded, not notched behind; (6) hindlimbs slender, heels overlapping and tibio-tarsal articulation reaching forward at the anterior corner of the eye; (7) tibia 0.47–0.51 of SVL and foot 0.64–0.69 of SVL in males, while tibia 0.44 of SVL and foot 0.51 of SVL in female; (8) fingers without lateral fringes, presence of distinct subarticular tubercles at the bases; (9) toes with lateral fringes and rudiment of webs, presence of subarticular tubercles at the bases; (10) presence of small horn-like tubercle at the edge of upper eyelid; (11) dorsal skin rough with sparse granules, a discontinuous X-shaped ridge with two discontinuous dorsolateral ridges on two side on the back; (12) sparse tubercles on the flanks; (13) orange-brown or light brown above, a dark interorbital triangle with light colored center and edge, a dark X-shaped making with light edge on the central of dorsum; (14) single subgular vocal sac in males; (15) presence of villiform black nuptial spines on the dorsal surface of the first and second fingers in adult males.

#### Comparison.

Comparative data of Megophrys (Panophrys) yangmingensis sp. nov. with M. (Pa.) mirabilis sp. nov., M. (Pa.) shimentaina sp. nov., M. (Pa.) xiangnanensis sp. nov., and 42 recognized congeners of *Panophrys* are given in Table 4.

Megophrys (Panophrys) yangmingensis sp. nov. differs from M. (Pa.) mirabilis sp. nov. by the smaller body size, SVL 33.2–37.1 mm in males and 45.2 mm in single female (vs. 55.8–61.4 mm in males and 68.5–74.8 in females), small horn-like tubercle at upper eyelid (vs. slightly large), presence of vocal sac in males (vs. absent), and presence of nuptial spines in adult males (vs. absent).

Megophrys (Panophrys) yangmingensis sp. nov. differs from M. (Pa.) shimentaina sp. nov. by the larger body size, SVL 33.2–37.1 mm in males (vs. 28.0–30.6 mm in males), absence of vomerine teeth (vs. present), and absence of tiny spines on the whole dorsal skin, flanks, dorsal limbs, the region around cloaca, and rear of hindlimbs (vs. such spines present).

Megophrys (Panophrys) yangmingensis sp. nov. differs from M. (Pa.) xiangnanensis sp. nov. by the smaller body size, SVL 33.2–37.1 mm in males (vs. 38.6–42.0), heels overlapping (vs. just meeting), tibio-tarsal articulation reaching forward at the anterior corner of the eye (vs. between eye and tympanum), and narrow lateral fringes on toes (vs. wide).

Compared with the five *Panophrys* species previously recorded from the hilly areas among Guangdong, Guangxi and Hunan, Megophrys (Panophrys) yangmingensis sp. nov. differs from M. (Pa.) acuta by the larger body size, SVL 33.2–37.1 mm in males and 45.2 mm in single female (vs. 27.1–33.0 mm in males and 28.1–33.6 mm in females), small horn-like tubercle at upper eyelid (vs. slightly large), snout rounded in dorsal view (vs. strongly remarkably pointed), and heels overlapping (vs. not meeting). M. (Pa.) yangmingensis sp. nov. differs from M. (Pa.) brachykolos by the narrow lateral fringes on toes (vs. absent), and heels overlapping (vs. not meeting). M. (Pa.) yangmingensis sp. nov. differs from M. (Pa.) nanlingensis by the absence of vomerine teeth (vs. present), and tongue not notched behind (vs. notched). M. (Pa.) yangmingensis sp. nov. differs from M. (Pa.) obesa by the narrow lateral fringes on toes (vs. absent), and heels overlapping (vs. not meeting). M. (Pa.) yangmingensis sp. nov. differs from M. (Pa.) shunhuangensis sp. nov. by the presence of vomerine ridge (vs. absence).

With a small body size, SVL 33.2–37.1 mm in adult males, Megophrys (Panophrys) yangmingensis sp. nov. is significantly different from nine congeners whose SVL > 40 mm in males, namely M. (Pa.) baolongensis, M. (Pa.) binlingensis, M. (Pa.) caudoprocta, M. (Pa.) jingdongensis, M. (Pa.) liboensis, M. (Pa.) omeimontis, M. (Pa.) sangzhiensis, M. (Pa.) shuichengensis, and M. (Pa.) spinata.

Megophrys (Panophrys) yangmingensis sp. nov. can be further distinguished from the remaining 28 congeners by the following characteristics: small horn-like tubercle at upper eyelid [vs. slightly large in M. (Pa.) jinggangensis, M. (Pa.) latidactyla, and M. (Pa.) palpebralespinosa]; vomerine teeth absent [vs. present in M. (Pa.) daweimontis, M. (Pa.) dongguanensis, M. (Pa.) fansipanensis, M. (Pa.) hoanglienensis, M. (Pa.) insularis, M. (Pa.) jinggangensis, M. (Pa.) jiulianensis, M. (Pa.) latidactyla, M. (Pa.) nankunensis, M. (Pa.) palpebralespinosa, and M. (Pa.) rubrimera]; tongue not notched behind [vs. notched in M. (Pa.) cheni, M. (Pa.) boettgeri, M. (Pa.) fansipanensis, M. (Pa.) hoanglienensis, M. (Pa.) huangshanensis, M. (Pa.) insularis, M. (Pa.) jiulianensis, M. (Pa.) kuatunensis, M. (Pa.) minor, and M. (Pa.) rubrimera]; lateral fringes on toes narrow [vs. absent in M. (Pa.) daweimontis, M. (Pa.) dongguanensis, M. (Pa.) fansipanensis, M. (Pa.) hoanglienensis, M. (Pa.) huangshanensis, M. (Pa.) insularis, M. (Pa.) jiangi, M. (Pa.) jiulianensis, M. (Pa.) leishanensis, M. (Pa.) lishuiensis, M. (Pa.) minor, M. (Pa.) nankunensis, M. (Pa.) ombrophila, M. (Pa.) tuberogranulatus, M. (Pa.) wugongensis, and M. (Pa.) wuliangshanensis; vs. wide in M. (Pa.) binchuanensis, M. (Pa.) boettgeri, M. (Pa.) cheni, M. (Pa.) latidactyla, M. (Pa.) lini, and M. (Pa.) palpebralespinosa; vs. absent in females while wide in males in M. (Pa.) wushanensis]; rudimentary webs on toes [vs. more than one-fourth webs in M. (Pa.) palpebralespinosa; vs. lacking webs in M. (Pa.) daweimontis, M. (Pa.) fansipanensis, M. (Pa.) hoanglienensis, M. (Pa.) huangshanensis, M. (Pa.) kuatunensis, M. (Pa.) lishuiensis, M. (Pa.) ombrophila, M. (Pa.) rubrimera, and M. (Pa.) wuliangshanensis]; tympanum clear with distinct edge [vs. upper 1/4 of tympanum concealed by supratympanic fold in M. (Pa.) mufumontana]; tibio-tarsal articulation reaching forward at the anterior corner of the eye [vs. between tympanum and eye in M. (Pa.) xianjuensis].

#### Description of holotype.

Adult male. Body size moderate, SVL 35.1 mm; head width slightly larger than head length, HDW/HDL 1.01; snout rounded in dorsal view, projecting, protruding well beyond margin of lower jaw; top of head flat; eyes large, ED 0.43 of HDL, pupil vertical; nostril oblique-ovoid; canthus rostralis well developed; loreal region slightly oblique; internasal distance slightly larger than interorbital distance; tympanum clear, TD/ED 0.43; large ovoid choanae at the base of the maxilla; presence of weak vomerine ridge, absence of vomerine teeth; margin of tongue rounded, not notched behind; presence of a single subgular vocal sac, a pair of slit-like openings at posterior of jaw.

Radio-ulna length 0.24 of SVL and hand 0.23 of SVL; hand without webs, fingers without lateral fringes, relative finger length II < I < IV < III; tips of fingers slightly dilated, round; one distinct subarticular tubercle at the bases of each finger; inner metacarpal tubercle observably enlarged and the outer one smaller; villiform black nuptial spines on the dorsal surface of the first and second fingers. Hindlimbs slender, tibio-tarsal articulation reaching forward at the anterior corner of eye when hindlimb is stretched along the side of the body; heels overlapping when the flexed hindlimbs are held at right angles to the body axis; tibia length 0.51 of SVL and foot length 0.67 of SVL; relative toe length I < II < V < III < IV; tips of toes round and slightly dilated; toes with lateral fringes and rudiment of webs; one subarticular tubercle at the bases of each toe; inner metatarsal tubercle long ovoid and the outer one absent.

Dorsal skin rough with sparse granules; sparse tubercles on the flanks and hindlimbs; several tubercles on upper eyelid, including a horn-like prominent tubercle on the edge; clear supratympanic fold curving postero-ventrally from posterior corner of eye to a level above insertion of arm; a discontinuous X-shaped ridge and two discontinuous dorsolateral ridges on two sides at the central back; four transverse skin ridges on the dorsal shank and thigh; ventral surface smooth; several granules on posterior hindlimbs; small pectoral gland closer to axilla; a single large femoral gland on rear of thigh.

#### Coloration.

Orange-brown above in life; a triangular making with light edge between eyes; a dark X-shaped making with light edge on the central of dorsum; supratympanic fold light brown; dark vertical band below the eye; iris orange-brown; throat and anterior chest purplish brown; belly dark gray with a large white blotch on the central; ventral limbs purplish; ventral hands reddish brown with dark stripes, tips of fingers pale-grey, metacarpal tubercle reddish; ventral feet purplish, tips of fingers pale-grey, metatarsal tubercle reddish; pectoral gland and femoral gland white.

#### Variations.

Measurement data of type series are listed in Table 8. All paratypes are similar to the holotype. The single female (SVL 45.2 mm) are distinctly larger than males (SVL 33.2–37.1 mm), while with relatively shorter hindlimbs. Dorsal surfaces lighter brown in SYS a002877, 2888–2889, 2891–2892.

#### Distribution and ecology.

Currently, Megophrys (Panophrys) yangmingensis sp. nov. is only known from Mt Yangming, southwestern Hunan. This toad inhabits near flowing mountain streams over 1300 m a.s.l. Males call from early June to early September. Males found in early June bear well developed nuptial spines, while the spines are absent in males found in early September, suggesting the breeding season of this toad is before September. Only one female was found, and tadpoles and more ecological information remain unknown.

#### Vocalization.

The advertisement calls of Megophrys (Panophrys) yangmingensis sp. nov. were recorded from the Holotype at 16 °C air temperature on 12 June 2014. Five calls with 160 notes are measured and the spectrograms are shown in Fig. 6C. The advertisement call is made up by 31.6 ± 9.0 (22–46, *N* = 5) continuous click notes. Each call lasts 7.38 ± 2.08 s (4.61–10.58 s, *N* = 5) and each note lasts 75 ± 5 ms (64–94 ms, *N* = 160) with an interval of 160 ± 31 ms (120–366 ms, *N* = 155) between every two notes. The peak frequency measures at 3424 ± 82 Hz (3375–3563 Hz, *N* = 160).

**Table 8.** Measurements (in mm) of the type series of Megophrys (Panophrys) yangmingensis sp. nov., * for the holotype.

	SYS a002887 *	SYS a002307	SYS a002310/CIB 116073	SYS a002888	SYS a002889	SYS a002891	SYS a002892	SYS a002877
**Sex**	Male	Male	Male	Male	Male	Male	Male	Female
**SVL**	35.1	34.5	36.6	33.2	37.1	36.4	34.5	45.2
**HDL**	11.3	11.6	11.7	11.2	11.9	11.5	11.2	13.6
**HDW**	11.4	11.9	11.7	11.1	11.8	11.5	11.3	13.5
**SNT**	4.0	4.4	4.2	3.8	4.4	4.3	4.2	4.8
**IND**	3.9	3.6	4.0	3.8	3.9	3.8	3.8	4.2
**IOD**	3.4	3.8	3.8	3.4	3.8	3.6	3.7	4.4
**ED**	4.9	4.7	4.6	4.8	4.7	5.0	4.7	5.6
**TD**	2.1	2.2	2.3	2.2	2.2	2.1	2.3	2.8
**TED**	1.5	1.4	1.4	1.4	2.1	1.7	1.6	2.1
**HND**	8.0	8.2	8.2	7.0	8.2	8.2	7.9	10.1
**RAD**	8.5	8.2	8.2	7.0	9.2	8.4	8.0	9.9
**FTL**	24.2	23.3	23.4	21.1	24.1	24.1	23.2	23.0
**TIB**	17.8	17.2	17.3	15.5	17.3	17.6	17.0	19.9

## Discussion

The phylogenetic analysis encompassing multilocus nuclear-gene and matrilineal mtDNA genealogy (Liu et al. 2018) has revealed 41 cryptic species within the subgenus Panophrys. Subsequently, eight of them were described as seven new species (Li et al. 2018; Wang et al. 2019a, b). It is worth noting that the cryptic species *M.* sp6 and *M.* sp7 revealed based on molecular data were suggested to be the same species and is described as M. (Pa.) nanlingensis after detailed morphological examination (Wang et al. 2019a). In our present study, we propose four new species, on the basis of detailed morphological evidences combined with previous phylogenetic data. There are 29 undescribed cryptic species remaining according to Liu et al. (2018), nevertheless, the recognitions from molecular data still require validation from detailed morphological characteristics to substantiate.

The genus *Panophrys* was established by Rao and Yang (1997) but was controversially considered as a subgenus or synonymy of *Xenophrys* or *Megophrys* by different subsequent morphological researches (Dubois and Ohler 1998; Delorme et al. 2006; Li and Wang 2008; Fei et al. 2009). Based on multilocus nuclear-gene and matrilineal mtDNA genealogy, three recent studies have revealed highly similar phylogenetic relationships within Megophryinae, which is unanimously considered to contain the following monophyletic groups: Pelobatrachus, Megophrys, Xenophrys, Panophrys, Brachytarsophrys, Ophryophryne, Atympanophrys (Chen et al. 2017; Mahony et al. 2017; Liu et al. 2018). However, the taxonomic proposals for these groups are in conflict by different authors. Chen et al. (2017) considered that subfamily Megophryinae is valid and composed of five genera: *Atympanophrys*, *Brachytarsophrys*, *Megophrys*, *Ophryophryne* and *Xenophrys* (including *Panophrys* as a subgenus). Mahony et al. (2017) treated the entire subfamily Megophryinae as a single genus *Megophrys* with containing seven subgenera (corresponding to the seven molecularly resolved clades). To resolve these conflicts, Li et al. (2020) suggested to elevate the seven monophyletic subgenera to genus levels, which fulfills the following three criteria to be descriptively useful: reasonably compact, monophyletic, and ecologically, morphologically or biogeographically distinct (Gill et al. 2005). Li et al.’s suggestion was based on the review of *Brachytarsophrys*, which shows significant differences against other groups. Therefore, the recognition of genus *Brachytarsophrys* must be accepted, while further supported evidences for other genera are needed.

## Supplementary Material

XML Treatment for Megophrys (Panophrys) mirabilis

XML Treatment for Megophrys (Panophrys) shimentaina

XML Treatment for Megophrys (Panophrys) xiangnanensis

XML Treatment for Megophrys (Panophrys) yangmingensis

## Appendix I

**Specimens examined**

Megophrys (Panophrys) acuta (10): **China: Guangdong**: Fengkai: Heishiding Nature Reserve (type locality): SYS a000168, 0517, 0521, 3257, 2159, 2266–2269, 2276.

Megophrys (Panophrys) binlingensis (2): **China: Sichuan**: Hongya: Mt. Wawu (type locality): SYS a005313–5314.

Megophrys (Panophrys) boettgeri (16): **China: Fujian**: Wuyishan: Mt. Wuyi (type locality): SYS a002480, 4149–4151; **Jiangxi**: Guixi: Yangjifeng Nature Reserve: SYS a000312, 0315, 0328–0330, 0376, 0378; Guangfeng: Tongboshan Nature Reserve: SYS a001671–1673, 1683, 1700.

Megophrys (Panophrys) brachykolos (2): **China: Hong Kong** (type locality): SYS a001502–1503.

Megophrys (Panophrys) caudoprocta (3): **China: Hunan**: Sangzhi: Badagongshan Nature Reserve (type locality): SYS a004281, 4308–4309.

Megophrys (Panophrys) cheni (19): **China: Jiangxi**: Jinggangshan: Mt. Jinggang (type locality): SYS a001427–1429, 1871–1873; **Hunan**: Yanling: Taoyuandong Nature Reserve: SYS a002123–2127, 2140–2145.

Megophrys (Panophrys) huangshanensis (13): **China: Anhui**: Huangshan: Mt. Huangshan (type locality): SYS a002702–2707; **Jiangxi**: Wuyuan: Mt. Dazhang: SYS a001622–1623, 3705–3707; **Zhejiang**: Lin’an: Mt. Tianmu: SYS a002684–2685.

Megophrys (Panophrys) jingdongensis (14): **China: Yunnan**: Jingdong: Mt. Wuliang (type locality): SYS a003909, 3928–3929; Zhenyuan: Mt. Ailao: SYS a001778, 2988, 2989–2991, 2993–2994, 3005–3006, 3903–3904.

Megophrys (Panophrys) jinggangensis (11): **China: Jiangxi**: Jinggangshan: Mt. Jinggang (type locality): SYS a001413–1416, 1430, 4028; **Hunan**: Yanling: Taoyuandong Nature Reserve: SYS a001859–1863.

Megophrys (Panophrys) kuatunensis (3): **China: Fujian**: Wuyishan: Guadun: SYS a001579, 1590; **Jiangxi**: Guixi: Yangjifeng Nature Reserve: SYS a000241.

Megophrys (Panophrys) lini (27): **China: Hunan**: Yanling: Taoyuandong Nature Reserve (type locality): SYS a002128; **Jiangxi**: Jinggangshan: Mt. Jinggang: SYS a001417–1424, 2375–2386; Suichuan: Nanfengmian Nature Reserve: SYS a002369–2374.

Megophrys (Panophrys) minor (5): **China: Sichuan**: Dujiangyan: Mt. Qingcheng (type locality): SYS a003209–3213.

Megophrys (Panophrys) obesa (4): **China: Guangdong**: Fengkai: Heishiding Nature Reserve (type locality): SYS a002270–2272, 3047.

Megophrys (Panophrys) omeimontis (11): **China: Sichuan**: Emeishan: Mt. Emei (type locality): SYS a001798–1801, 1940–1941, 5301; Hongya: Mt. Wawu: SYS a005330–5331; Pingshan: Mt. Laojun: SYS a002740–2741.

Megophrys (Panophrys) sangzhiensis (6): **China: Hunan**: Sangzhi: Badagongshan Nature Reserve (type locality): SYS a004306–4307, 4313–4316.

Megophrys (Panophrys) spinata (2): **China: Guizhou**: Leishan: Mt. Leigong (type locality): SYS a002226–2227.

Megophrys (Panophrys) tuberogranulatus (1): **China: Hunan**: Sangzhi: Badagongshan Nature Reserve (type locality): SYS a004310.

Megophrys (Panophrys) wushanensis (5): **China: Hubei**: Shennongjia: Shennongjia Nature Reserve: SYS a003008–3011, 3013.

Megophrys (Panophrys) wuliangshanensis (5): **China: Yunnan**: Jingdong: Mt. Wuliang (type locality): SYS a003924–3925; Zhenyuan: Mt. Ailao: SYS a002983–29

Megophrys (Xenophrys) glandulosa (13): **China: Yunnan**: Jingdong: Mt. Wuliang (type locality): SYS a003907–3908, 3923; Tengchong: Gaoligong Nature Reserve: SYS a002944–2946, 3757–3758, 3762, 3792–3795.

Megophrys (Xenophrys) cf. major (3): **China: Yunnan**: Mengla: Zhushihe: SYS a002961–2962, 3955.

Megophrys (Xenophrys) mangshanensis (10): **China: Guangdong**: Ruyuan: Nanling Nature Reserve: SYS a000493–0496, 0586; Renhua: Danxiashan Geological Park: SYS a000288; Huaiji: Mt. Sanyue: SYS a002177; Shaoguan: Mt. Longtou: SYS a002749; **Jiangxi**: Longnan: Jiulianshan Nature Reserve: SYS a000996–0997.

Megophrys (Xenophrys) medogensis (3): **China: Xizang**: Motuo: Beibeng (type locality): SYS a002932–2933, 2935.

Megophrys (Xenophrys) pachyproctus (1): **China: Xizang**: Motuo: Beibeng (type locality): SYS a002934.
